# Label-free optical biosensors in the pandemic era

**DOI:** 10.1515/nanoph-2022-0354

**Published:** 2022-08-12

**Authors:** Giovanni Nava, Giuliano Zanchetta, Fabio Giavazzi, Marco Buscaglia

**Affiliations:** Dipartimento di Biotecnologie Mediche e Medicina Traslazionale, Università degli Studi di Milano, Segrate, MI, Italy

**Keywords:** digital biosensors, DNA nanotechnology, rapid analytical methods, single-molecule detection, ultrasensitive biosensors, whole virus detection

## Abstract

The research in the field of optical biosensors is continuously expanding, thanks both to the introduction of brand new technologies and the ingenious use of established methods. A new awareness on the potential societal impact of this research has arisen as a consequence of the Covid-19 pandemic. The availability of a new generation of analytical tools enabling a more accurate understanding of bio-molecular processes or the development of distributed diagnostic devices with improved performance is now in greater demand and more clearly envisioned, but not yet achieved. In this review, we focus on emerging innovation opportunities conveyed by label-free optical biosensors. We review the most recent innovations in label-free optical biosensor technology in consideration of their competitive potential in selected application areas. The operational simplicity implicit to label-free detection can be exploited in novel rapid and compact devices for distributed diagnostic applications. The adaptability to any molecular recognition or conformational process facilitates the integration of DNA nanostructures carrying novel functions. The high sensitivity to nanoscale objects stimulates the development of ultrasensitive systems down to digital detection of single molecular binding events enhanced by nanoparticles and direct enumeration of bio-nanoparticles like viruses.

## 1 Introduction

Biosensors are devices capable of converting bio-molecular binding events into detectable and quantifiable physical signals. In label-free optical biosensors, the signal is generated directly by the local change of refractive index upon binding of a target analyte to the recognition element, typically immobilized on a surface, without requiring additional labelling agents, be they colorimetric, fluorescent or chemiluminescent [1]. Label-free biosensors provide potential advantages in terms of simplicity and rapidity of the measurement and enable accessing the kinetics of the molecular recognition process. The increasing number and diversity of systems that can be defined as optical biosensors make it difficult to orient oneself in this area despite the tremendous potential impact on both bio-molecular research and diagnostic applications [2]. In this scenario, the worldwide emergency caused by Covid-19 pandemic has clearly shown the high societal impact of the availability of quantitative analytical tools to investigate the complex behaviour of biological elements from small molecules up to cells and tissues, as well as of diagnostic systems to efficiently monitor the spread of the infection and the related health conditions [3]. However, despite the abundance of innovative technologies, their impact on the global fight against the SARS-CoV-2 pandemic has been very limited, and most of the analytical tools effectively exploited were still based on standard approaches [4]. Covid-19 emergency made clear the challenges in developing novel reliable and widely useable diagnostic assays or research instruments based on the most advanced biosensor technologies [5]. The development of systems that are at the same time sensitive and reliable requires several features that are typically difficult to combine. On one side, the awareness on the societal benefit of improving the current standards has increased, also because of the ever increasing frequency of outbreaks [6]; on the other side, innovative technologies have to meet an expanded set of requirements and benchmarks. Biosensors based on label-free optical transduction of the signal due to molecular recognition events are good candidates for next generation technologies for bio-analysis, because they enable the real-time observation of biomolecular binding and potentially minimize the sample processing steps.

**Figure 1:**
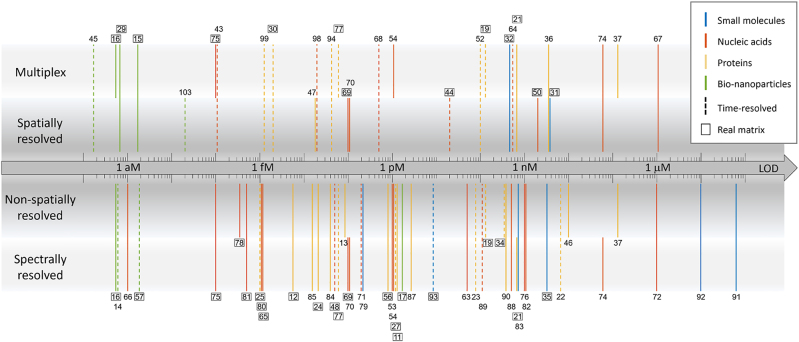
Graphical table summarizing the references considered in this review reporting a value of LOD. Reference numbers are associated to vertical segments indicating the value of LOD on the horizontal axis and the class of biosensor method on the vertical axis. The inner classifications (spatially resolved or non-spatially resolved methods) are mutually exclusive, whereas systems assigned to different combinations of the outer classes (multiplex and spectrally resolved methods) are reported. The segment colour indicates the type of target according to the legend. Dashed line segments indicate the methods exploiting time-resolved capabilities of label-free acquisition. Outlined reference numbers indicate the detection methods demonstrated in real matrices (e.g. blood or saliva).

In a previous review [7], we highlighted some of the most promising emerging applications of optical label-free biosensors and grouped them according to the type of target analyte, from small molecules to bio-nanoparticles, and the class of sensing substrate, either planar or nanostructured and either metallic or dielectric. In this review, we address the recent advances in the field of label-free optical biosensors by focusing on their expected potential to provide an impact as either research or diagnostic tools in the post-pandemic world. A synthetic view of the wide range of capabilities of label-free optical biosensors is given in Figure 1, which collects results from the papers included in this review, for which it was possible to define a value for the limit of detection (LOD) for a relevant analyte. Each work is represented by one or more vertical segments, as indicated by the reference numbers. The horizontal axis of the figure shows that different biosensor formats can span an extremely large range of LOD, down to attomolar concentrations. In particular, the region of LOD below 1 pM in recent years is increasingly populated with different innovative biosensor designs, which can be defined as ultrasensitive. The vertical distribution in Figure 1 indicates different classes of sensing methods. First, we choose to distinguish between spatially-resolved and non-spatially resolved approaches, because they typically differ in terms of optical design and processing of the signal. Additional categories indicated in Figure 1 are multiplex capabilities, usually associated to spatially-resolved methods, and spectral discrimination, usually combined to non-spatially resolved methods, although different combinations of all the four categories can be present. The colour of the segments of Figure 1 represents the type of target analyte, from small molecules to proteins, nucleic acids (NA) and bio-nanoparticles (e.g. exosomes or viruses). Methods providing kinetic analysis enabled by time-resolved acquisition are indicated as dashed segments. Additionally, the reference number of the papers, in which the biosensors have been demonstrated with real matrices like blood or saliva, are outlined. Quite surprisingly, all these features seem almost equally distributed along the LOD scale at glance. As expected, methods addressing the detection of small molecules tend to reach larger LOD, whereas in the attomolar region most of the methods address much larger bio-nanoparticle targets.

In the following Sections, recently proposed, selected optical label-free biosensors are discussed according to five classes of potential applications: biosensor-based analytical systems for rapid, decentralized human diagnostics (Section 2); biosensor schemes exploiting DNA nanostructures for target capture or signal enhancement (Section 3); detection methods relying on the use of nanoparticles (NP), either for nanostructures fabrication or for signal amplification, down to ultrasensitive detection of biomolecules (Section 4); biosensors enabling direct enumeration of single viruses or exosomes (Section 5); methods achieving single molecule detection capability (Section 6).

## 2 From research laboratory to routine diagnostics

Label-free biosensors are commonly used in research laboratories to quantitatively assess molecular interaction parameters thanks to their ability to provide direct access to kinetic binding curves, which is an irreplaceable feature for this class of application. In contrast, exploitation of label-free biosensors for detection and quantification of pathogens or biomarkers in biological fluids for diagnostic applications remains sporadic [8]. In principle, biosensor-based diagnostic tools can provide simpler protocols suitable for decentralized settings, direct and fast quantification and miniaturization of the device. These features are common goals of the research aiming at developing innovative diagnostic approaches. However, other limitations, not necessarily related to the biosensor design, but rather to the lower maturity of development relative to established standard methods, have so far prevented the widespread use of label-free biosensors for routine diagnostics, including robustness, repeatability and scalability of cost and production. A potential gain of competitive positioning can be sought in those features that enable novel kind of diagnostic analysis, not suitably covered by current methods. In this framework, the experience acquired during the pandemic response represents an unprecedented milestone providing extensive validation of current state of the art of diagnostic systems and making clear the main needs to be addressed by future development in this area. Examples of the research effort aimed at developing label-free optical biosensors specifically designed to contribute to the new challenges emerged by Covid-19 pandemic experience are presented below (Section 2.1). Among the many biosensor designs proposed in the scientific literature, we further focused on two alternative formats potentially suitable for decentralized diagnostics and offering explicit advantages relative to current standards (Section 2.2): biosensors based on optical fibers and image-based biosensors. Another potential advantage given by the use of label-free biosensors as diagnostic systems can be sought in compact device formats suitable for portable systems (Section 2.3). A selection of recent work providing promising advancements in these categories of biosensors is described in the following Subsections.

### 2.1 Challenges of the pandemic response

The diagnostic methods that had more impact in the fight to the Covid-19 pandemic have concerned the detection of viral RNA or spike proteins in oropharyngeal swabs by real-time polymer chain reaction (RT-PCR) or lateral flow device (LFD), respectively [4]. These targets are used primarily to discriminate between infected and non-infected subjects and secondarily as a quantification of the level of infection. Another class of common analytical targets is provided by antibodies against SARS-CoV-2, whose presence, detected in serological tests, indicates a previous exposure to the virus and to some extent can provide a measure of the current protection towards future infection, although the simple quantification of IgG is not found to correlate well with the symptoms of Covid-19 [9]. Since the first stages of the pandemic, the research on label-free optical biosensors has directed considerable efforts in the quest for novel diagnostic methods providing some beneficial advancement relative to the standard approaches. Given the intrinsic flexibility of the label-free design, potentially all diagnostic targets can be addressed by this class of biosensors. In particular, nucleic acids, viral proteins and antibodies have all been proposed as targets of possible diagnostic tools [10]. Additionally, specific biosensor formats can be exploited to quantify analytical targets not currently within the reach of standard methods, such as the amount of whole virus particles, which can be more directly associated to the contagiousness of infected subjects.

The detection and quantification of viral RNA by RT-PCR in centralized laboratories currently is one of the most reliable and sensitive diagnostic approaches, largely exploited in the fight against SARS-CoV-2. The research on optical biosensors targeting nucleic acids brings the expectations of more rapid and simple systems suitable for distributed diagnostic solutions, while aiming to maintain extremely low LOD of a few copies/mL typical of RT-PCR. Indeed, this direction of development also takes advantage from the results of the expanding field of DNA-nanotechnology, as further discussed in Section 3. In contrast, the detection of protein targets by standard methods such as ELISA or LFD typically reaches larger LOD, in the pM to nM range. In this context, label-free biosensors with improved sensitivity have been proposed as promising alternative methods. As an example, long-period fiber grating has been demonstrated for the detection of SARS-CoV-2 spike protein down to 100 pg/mL concentration [11]. The design of the fiber grating enables to compensate for environmental temperature, hence increasing the robustness of the method. Ultrasensitive biosensors with LOD below the pM range are emerging as solutions with disruptive potential in recent work. The signal obtained by imprinted photonic crystal film and acquired by a smartphone enabled the detection of about 430 fg/mL of spike protein in saliva [12]. Nanoparticle-enhanced surface plasmon resonance (SPR) has been exploited for the detection of as low as 85 fM of nucleocapsid protein in buffer by a sandwich assay format [13].

A promising alternative to ultrasensitive detection of viral antigen is provided by novel methods enabling the direct enumeration of whole virus particles down to quantities comparable with RT-PCR sensitivity. SAR-CoV-2 virions display a diameter of about 100 nm, hence below the diffraction limit of light. However, they can be imaged as diffraction-limited points and enumerated by optical microscopy, if the signal-to-noise ratio is made large enough. In this context, biosensor substrates can provide the required enhancement of the optical signal upon binding of virions. The detection of pseudo-virus particles by just a plate reader or a smartphone has been demonstrated by nanoplasmonic biosensor enhanced by gold nanoparticles (AuNP) [14]. The enumeration of intact virion particles in saliva has been reported in a few studies based on different biosensor designs: interferometric scattering microscopy on a photonic resonator surface has been demonstrated to enable the detection of single virions down to concentrations of 1000 particles/mL [15]; a localized SPR (LSPR) system realized by nanoporous gold on a SiO_2_ vertical nano-cavity structure provided the rapid detection of about 300 particles/mL of virions [16]; another promising approach, although currently yielding a much larger LOD, is provided by the combination of machine learning algorithms with signals from surface-enhanced Raman spectroscopy (SERS, of which we consider here only genuine label-free implementations), measured through metal–insulator–metal nanostructures fabricated on flexible substrates [17].

In contrast to antigen or virion detection, for serological testing the common LOD of standard methods are perceived as adequate to the typical concentrations of antibodies in blood. However, improvements are sought in novel devices capable of conjugating large sensitivity, operational simplicity, rapidity and portability. Cost effective biosensors substrates based on interferometric optical signal from SiO_2_ thin layer on Si substrates have been proposed to quantify antibodies in serum and saliva showing a good correlation with quantification performed by ELISA [18]. Sensitive and rapid detection of IgG in serum has also been demonstrated by a SPR-based portable device employing a multiantigen sensor biochip functionalized with RBD peptide and N protein, reaching a LOD of about 20 ng/mL and a measuring time below 15 min [19]. A serological profiling based on the binding kinetics of IgG in serum and whole blood on immobilized RBD was achieved by a fiber optic SPR system with a time-to-result of about 30 min [20]. A nanoplasmonic biosensor providing a multiplexed spectroscopic signal upon binding of antibodies on SARS-CoV-2 spike and nucleocapsid proteins has been demonstrated for serological profiling in low volumes of serum [21]. A novel and promising approach for IgG detection, potentially very sensitive although currently demonstrated only in the μg/mL range, is based on a disposable whispering gallery mode (WGM) sensor realized by self-assembled silica microspheres [22].

### 2.2 Fiber-based and image-based label-free biosensors for point-of-care diagnostics

Many novel formats of optical label-free biosensors suitable for distributed diagnostic applications have been proposed in recent years. Among these, we selected two main classes of designs, which offer promising features, typically not provided by current standard methods. In the case of biosensors based on optical fibers, the sensing surface is immersed directly into the specimen, hence enabling a rapid and local testing of different samples for the same target analyte without a specific measuring cell to be loaded and without a pumping or mixing system. Differently, image-based designs offer the potential advantage of multiplexing, hence enabling testing the same sample for different analytes in a single measurement.

#### 2.2.1 Fiber-based biosensors

**Figure 2:**
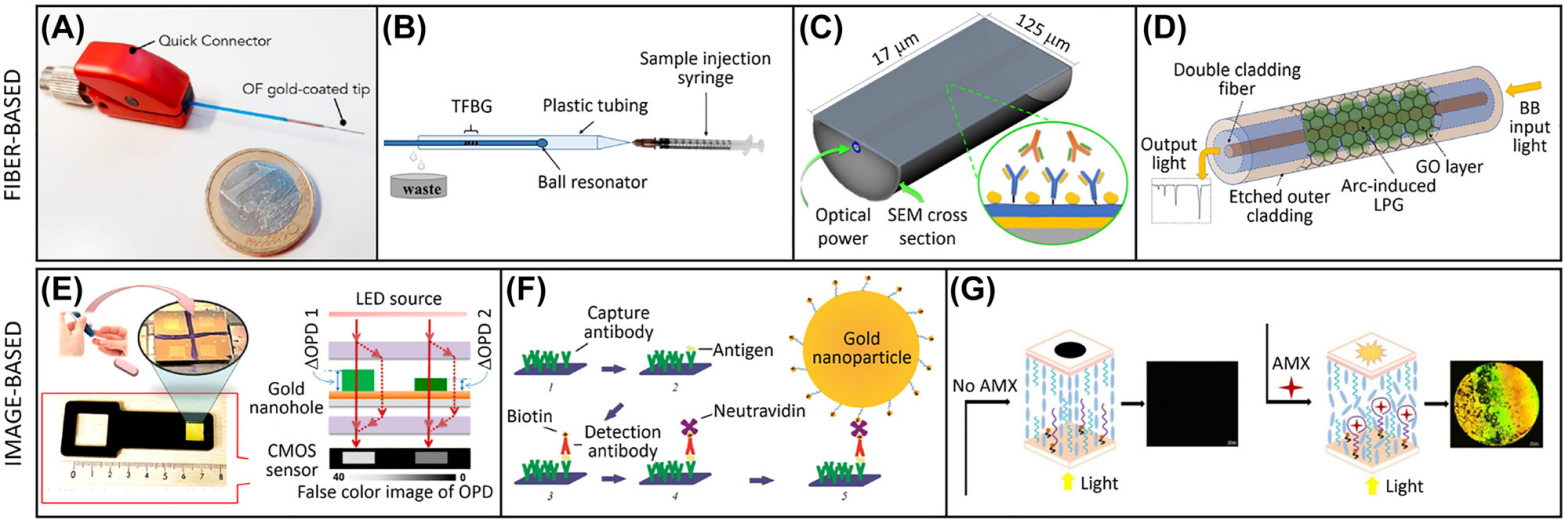
Fiber-based and image-based label-free biosensors as tools for point-of-care diagnostics. (A) SPR sensing on the tip of a fiber. (B) Fiber ball-tip resonator assisted by fiber Bragg grating. (C) D-shaped fiber lossy mode resonator. (D) Long period grating fiber coated with polycarbonate and graphene oxide. (E) Interferometric imaging of nanoplasmonic microarrays. (F) SPRi enhanced by AuNP though a sandwich assay format. (G) Liquid crystal biosensor for the detection of antibiotic. Reproduced with permission from: [23] (A), [24] (B), [25] (C), [27] (D), [28] (E), [29] (F), [30] (G).

Ultraminiature sensors can be realized by modifying the facet or the tip of optical fibers, as further discussed below in Section 4. In the contest of point-of-care (POC) testing, very low LOD were demonstrated for the detection of cancer biomarkers, an application in which the sensitivity of detection can be particularly relevant. A SPR sensing surface realized on the tip of an optical fiber enabled the detection of breast cancer biomarker HER2 with a LOD or 77 pM [23] (Figure 2A). The same biomarker was detected down to fg/mL in buffer and pg/mL in serum using a fiber ball-tip resonator assisted by tilted fiber Bragg grating [24] (Figure 2B). A different class of fiber-based sensors relying on lossy mode resonance was obtained by a D-shaped fiber. A device based on this concept enabled to measure IgG in serum down to fM concentrations [25] (Figure 2C). Larger LOD, although still competitive with standard methods, were obtained by long period grating fiber coated with polycarbonate and graphene oxide. Despite ultrasensitive detection was achieved for avidin–biotin binding, a model interaction with exceptionally large affinity [26], a similar device based on antibody-antigen recognition enabled the detection of c-reactive protein in serum with a LOD of 0.15 ng/mL [27] (Figure 2D).

#### 2.2.2 Image-based biosensors

The acquisition of biosensor signals spatially resolved in 2D gives access to a large amount of information that can be potentially exploited to measure different interactions in parallel and to further refine the analysis by taking advantage of reference and control signals or by morphological constraints. The potentiality of image-based systems has been increasing thanks to the growing availability of hardware components and algorithms enabling real-time execution of complex processing. Plasmonic or interferometric biosensor surfaces are often used to achieve image-based label-free detection. SPR imaging (SPRi) was used to demonstrate the detection of exosomes of neural origin in blood at a level of sensitivity of 1 μg/mL, similar to standard methods based on ELISA, but with the advantage of simultaneous analysis of multiple subpopulations [31]. The analysis of interferometric images from nanoplasmonic microarrays enabled rapid quantification of bacteria in clinical settings in order to identify sepsis patients [28] (Figure 2E). Ultrasensitivity and digital detection can be reached by further enhancement through secondary binding with NP. The quantification of cytokines in synovial fluid down to 50 fg/mL was demonstrated by SPRi enhanced by 40 nm AuNP though an antibody sandwich assay format [29] (Figure 2F). A different class of biosensors relies on the optical response of liquid crystal materials upon formation of specific probe-target complexes. Although the current LOD of these systems are typically still large, the potential simplicity of the device and the intrinsic amplification effect of molecular phenomena through macroscopic changes of the liquid crystal alignment makes them a promising solution for POC applications. Examples of biosensors based on this concept and using aptamers (folded DNA single strands which specifically bind a target molecule) as probes were demonstrated for the detection of amoxicillin antibiotic [30] (Figure 2G) and of pesticides [32] in environmental water.

### 2.3 Compact diagnostic devices

The integration of both bio-recognition elements and signal transduction mechanisms typical of the biosensor substrates facilitates the design of compact devices suitable for POC diagnostics. Many portable analytical devices embedding label-free optical biosensors have been proposed in the literature. A large portion of these exploit smartphones as acquisition, processing and communication modules [33]. This class of portable diagnostic devices is particularly suitable for rapid although accurate testing in resource-limited settings. As an example, a recently proposed approach relies on a gold-sputtered U-bent plastic optic fiber functionalized with an aptamer capturing a malaria biomarker and coupled to a smartphone providing illumination and detection. This device enabled to reach a LOD of about 350 pM of *Plasmodium falciparum* glutamate dehydrogenase in serum samples in about 20 min [34]. Another application, in which the availability of rapid and sensitive POC is particularly relevant is the diagnosis of sepsis. An example of proof-of-concept device for this application is provided by a grating-coupled SPR system conjugated with a smartphone for the detection of lipopolysaccharides, one of the main constituents of the outer membrane of Gram-negative bacteria that can trigger septic shock by inflammatory stimulation. In this case, lipopolysaccharides spiked in intravenous infusion liquids were detected with a LOD of about 30 ng/mL after 20 min incubation [35]. Other compact biosensors have been demonstrated as proof of principle with model interactions, although not yet reaching competitive LOD. We mention a couple of examples of promising designs that in principle could be further improved to reach lower LOD. A plasmonic nanohole microarray was tested for IgG detection enabling the simultaneous reading of hundreds of spots by custom interferometric lens-free imaging device with large field-of-view [36]. The fluidic handling of samples and reagents is elegantly addressed without the use of pumps by designs based on spinning disk micro-fluidics. An example of this class of devices embedded plasma extraction and aliquoting from whole blood and provided multiplex SPR detection through a smartphone camera [37]. Also this system was demonstrated with an interaction between anti-IgG and IgG although with large LOD.

## 3 Nucleic acids: tough targets, flexible and constructive probes

Because of their central role in biology, in human physiology and pathology, NAs are often a key target for diagnostic purposes, like in the cases of the quantification of circulating microRNAs (miRNAs) as biomarkers for several diseases [38] or the detection of genomic DNA or RNA of pathogens [10]. NA are often elusive and challenging targets, because of low concentration, instability of RNA, and numerous possibilities of non-specific binding arising from sequence variability. On the other hand, the same NA modular structure and the corresponding diversity of tridimensional shapes offer endless opportunities of using NA as nanometer-scale building blocks and to optimize the performance of NA probes [39]. For this reason we decided to devote a specific section to this class of biomolecules. Covering the entire, rapid development of the field in the last few years goes beyond the scope of this article. Its application to optical label-free biosensors has been partially covered by some recent reviews [10, 38, 40, 41]. Rather, we will present here a few interesting examples, suggesting emerging directions for NA as probes and as targets. They may seem mostly technical improvements and optimization of existing approaches, or application of existing techniques to novel targets. However, they witness an ever growing, creative use of the intrinsic versatility of NA for tunable secondary and tertiary structures. Moreover, they demonstrate a growing understanding of the details of the interactions which determine the biosensing performance.

### 3.1 Fishing with nucleic acids

Far from being limited to the role of linear probes with sequence complementarity to linear targets, NAs are literally flexible biomolecules which can adopt a variety of conformations and interact with myriad other molecules. Furthermore, NA base pairing interactions can be exploited to design folding and self-assembling 2D and 3D nanoconstructs, with applications also in the biosensing field.

#### 3.1.1 Building and sensing with DNA

**Figure 3:**
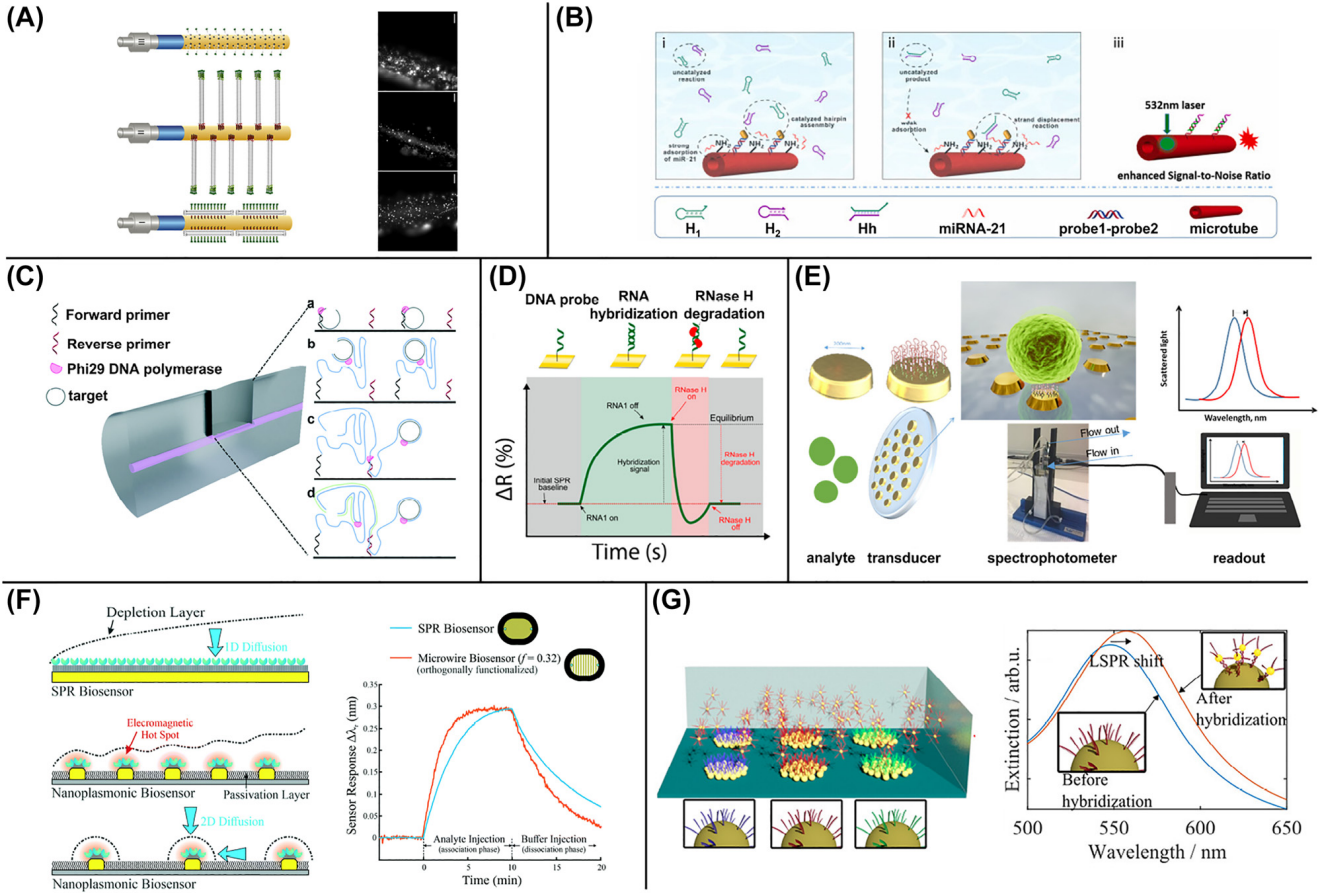
Nucleic acids as versatile probes and challenging targets. (A) An optical fiber-SPR biosensor is functionalized with DNA origami and nanostructures on the lateral surface or on the tip, to control the position of an aptamer. Fluorescently labeled oligonucleotides demonstrate the immobilization of DNA nanostructures. (B) Binding of miRNAs triggers catalytic hairpin assembly on the surface of a waveguide and minimizes background signals. (C) Upon binding of a circular DNA target in a microcavity, rolling circle amplification is triggered and monitored over time. (D) On a SPR chip, detection of hybridization is followed by RNase H degradation, exploited for alternative splicing analysis. (E) Schematic design of a portable LSPR platform for aptamer-based capture of bacteria. (F) The geometry of a nanoplasmonic surface determines the shape of the depletion layer and thus the rate of target DNA transport, as shown in the right panel for plain SPR versus microwires. (G) Gold nanoparticles, immobilized on glass substrates and functionalized with DNA strands, act as LSPR substrates displaying a peak shift upon hybridization. Reproduced with permission from: [46] (A), [48] (B), [49] (C), [50] (D), [51] (E), [52] (F), [53] (G).

The burgeoning field of DNA nanotechnology, in which DNA acts as a molecular building block, is proving increasingly useful for biosensors, as it allows controlling probe position and distance from the surface with nanometer resolution, dynamically tethering probes and nanoparticles, or amplifying binding signals. It thus offers unprecedented possibilities and is well suited for the real-time capabilities of label-free optical techniques [39, 42]. In a simple yet critical role, DNA strands can act as flexible connectors between a biosensing surface and functionalized nano- or microparticles, whose mobility and distance from the surface can be quantitatively correlated with binding events [43, 44], as will be discussed in Section 4. More in general, DNA strands can mediate the surface-immobilization of DNA-conjugated antibodies specific for e.g. viruses, which were detected via IRIS [45]. This modular approach, besides its versatility, has the advantage of keeping antibodies relatively far from the surface and more available for binding. DNA bundle-shaped origami were designed to functionalize a fiber-based SPR biosensor and to tune the mutual distance and 3D orientation of probes, to optimize the binding of human thrombin [46], as shown in Figure 3A. DNA origami were also used to tune the space distribution of receptors for a peanut antigen on the surface of a magnetic microparticle [47]. The binding was then detected and amplified with antibodies in a digital assay scheme in a multiwell. The nanopositioning resulted in a stronger, polyvalent interaction and lower LOD than with a single receptor. Amplification can also be obtained directly from self-assembly of DNA strands, as reported for example in [48] for a microtube waveguide biosensor: detection of miRNA in human serum triggered the so-called Catalytic Hairpin Assembly, in a novel design which reduced false positives, as shown in Figure 3B.

Optical antennas can be created by keeping at close distance two metal nanoparticles, with a fluorescent molecule in the gap, whose emission is amplified and tuned by the surface plasmons of nanoparticles [42]. A DNA origami was used to position a single fluorescent molecule in the optical antenna with nanometer control, which allowed characterizing the dipolar emission pattern [54]. Such field enhancement was exploited for single molecule detection, with a DNA origami construct keeping together two silver nanoparticles and capturing a target strand in the hotspot on the nanoantenna [55]. The exceptionally bright emission of an imager strand then allowed the detection of single strand binding events with a smartphone-based microscope.

#### 3.1.2 DNA catches them all: non-DNA targets

Due to their importance in biological mechanisms, NAs are also involved in countless interactions with other biomolecules. As such, the power of NA as biosensing elements also lies in their possible uses as a probe. Paradigmatic examples come from NA interactions with proteins like transcription factors or enzymes, whose activity is intrinsically related to binding and unbinding events, often with sequence selectivity. Therefore, the access to kinetics of label-free methods like SPR, reflective phantom interface (RPI) or biolayer interferometry [56] is particularly important to gain a full picture. For example, in [57], binding of the transcription factor Gal4 to do DNA double strands, with or without the consensus sequence, was investigated by RPI at various ionic strengths and temperatures. From the combined analysis a subtle interplay emerged between non-specific interactions, dominated by electrostatics, and conformational freezing associated with the formation of specific DNA-protein hydrogen bonds. A particular class of DNA binding proteins is constituted by isothermal polymerases like phi29, which can replicate a circular template into very long single strands, at high speed and fidelity. With a fiber-based Mach–Zehnder interferometer in a microcavity, filament elongation was measured over time [49], as sketched in Figure 3C. Conversely, the activity of a sequence-specific RNase, cleaving long mRNA filaments into smaller strands, was monitored over time with SPR [50] (Figure 3D), which in turn allowed to characterize the occurrence of alternative splicing.

One of the most spectacular manifestations of NA versatility is provided by aptamers, which are identified from an initial large, random pool of sequences through numerous selection cycles against the target. Despite the non-trivial, ever improving, selection procedure [58], because of their simple production and the possibility to easily immobilize them on a surface, aptamers are finding widespread use as probes in optical biosensors against several different targets. For example, an array of gold nanoparticles for SERS was functionalized with DNA strands containing an aptamer for interleukin-6 and a reporter tract [59]; its proximity to the substrate changed upon DNA folding and target binding, which in turn affected the Raman signal. A portable label-free optical sensor based on LSPR and aptamer recognition was developed for fast detection of *Staphylococcus aureus* in milk [51]. Filaments were immobilized on a nanostructured substrate with gold nanodisks (Figure 3E) and demonstrated a better performance than control antibodies, possibly because of closer proximity to the surface. Results were validated through fluorescence microscopy. It is worth mentioning that also for SARS-CoV-2 and other respiratory viruses an increasing number of aptamers are becoming available, including innovative approaches to COVID-19 treatment and targeting ACE2 receptor [60]. Such aptamers bind either the receptor binding domain [61] or other parts of the Spike protein [62] with nanomolar affinity. This may enable optical label-free approaches to give a substantial contribution to rapid diagnostics of viruses involved in respiratory infections [10].

### 3.2 Challenges of NA-based biosensors

#### 3.2.1 Fight for the real kinetics

When NA strands are attached to a biosensing surface as probes to capture complementary target strands in solution, several factors may affect their mutual interaction and thus the accurate estimate of their binding affinity and kinetics. These include, for example, probe–probe and probe–surface interfering interactions, which are exacerbated by NA linear structure. In particular, different surface chemistries and probe immobilization strategies can strongly influence their stability and accessibility and the degree of non-specific binding [63, 64]. Moreover, the transport properties of target molecules in proximity of the substrate can significantly alter the measured kinetic rates. For example, different shapes and surface density of nanoplasmonic patterns, like microwires, nanodisks and nanorods, led to different binding kinetics (besides the expected differences in signal enhancement) by SPR [52]. As shown in Figure 3F, while an effective 1D diffusion of targets occurs for a planar surface, access to probes is faster in the case of microwires because of the effective 2D diffusion. Ionic strength can also strongly influence kinetics [65]: as negatively charged target strands accumulate on the surface upon hybridization of the complementary probes, a layer above is depleted by electrostatic repulsion, which slows down further binding. This effect and its dependence on surface density can be well described in terms of an electrostatic penalty, allowing retrieving the real thermodynamic quantities and providing guidance for the design of assays.

#### 3.2.2 Towards real settings

Alternative strategies to enhance sensitivity are constantly seeked. For example, local field is enhanced on SPR chips with graphene oxide [66] and more recently through antimonene, another 2D material with appealing optical properties [67]. A competitive assay was developed, based on differential binding on antimonene of single- and double-stranded DNA, coupled to metal nanorods. Release of the molecules from the surface was detected by SPR, providing aM sensitivity. How would these numbers change from well-controlled situations to more realistic settings remains an open question. More in general, bringing label-free optical methods to the field clearly requires several additional challenges to be addressed, as also discussed in Section 2. In the context of the detection and quantification of nucleic acids, these arise from e.g. the complexity of biological fluids, like capturing miRNA in plasma [38, 41], and the need for simple and fast measurements, which often requires further amplification steps. In [68], a biosensor based on lens-free microscopy and light interferometry, with a gold nanohole array functionalized to capture a panel of biomarkers for early diagnosis of sepsis, including a miRNA target, provided fast response but only moderate LOD. In [69], label-free characterization of each assay step by RPI allowed the optimization of relative concentrations and time intervals, leading to a homogeneous assay for miRNA with sub-pM LOD in less than 2 h. However, the use of a primary antibody, targeting DNA-RNA hybrids, and of a secondary antibody was necessary. The other critical requirements of simplicity and reusability of devices was addressed in [70]: an interchangeable chip was mounted on a microfluidic cell, functionalized with molecular beacons to enhance SERS signal for miRNA detection, demonstrating sub-pM LOD in serum. In another portable SERS platform, with NA probes grafted on a gold grating [71], different spectra for complementary, mismatched and non-specific sequences were analyzed through principal component analysis to extract a synthetic, quantitative signal sensitive to sub-pM target concentration (sub-nM in presence of BSA). However, the origin of spectral changes even in absence of complementary sequences is not clear.

In the case of COVID-19 and other respiratory infections [10], innovative solutions are being proposed for the accurate detection of genomic sequences. Local heating, produced by plasmonic photothermal effect on gold nanoislands in a LSPR device, was found to accelerate kinetics and improve specific target capture in a mixture of similar sequences [72].

## 4 Nanoparticle-assisted target detection

Analytical methods assisted by NP are nowadays covering a large part of the biosensing market, because NP are a cost-effective, everlasting, robust tag, whose biochemical (surface modifications with antibodies, proteins, nucleic acids) and physical properties (refractive index, size, plasmonic effects) can be precisely tailored for specificity or amplification purposes and yet highly reproducible at the same time. Moreover, NP are widely employed in surface-based optical biosensors for a twofold purpose: (i) the immobilization of NP can provide a convenient strategy to fabricate nanostructured sensing surfaces with controlled roughness and functionality, either through direct deposition of metallic NP, or by using dielectric colloids as masks for metallic layers; (ii) the binding of a single NP mediated by a molecular interaction can provide enough optical signal to enable digital, NP-enhanced detection. NP-assisted detection schemes meet naturally the growing need of the transition from an integral continuum detection regime to a digital counting regime. A transition to a single event counting is mostly driven by strict diagnostic requirements that in some cases (see next paragraph and Section 2) push the LOD for specific analytes below 1 pM.

Biosensors based on NP-assisted fabrication (i) or NP-assisted detection (ii) mostly correspond to two main types of output signals: spectrally resolved and spatially resolved biosensors.

### 4.1 Spectrum resolved NP-assisted detection

The expected response of the NP to light is the central aspect of the detection schemes; most of the technologies here presented rely on the interplay between the plasmonic properties of the NP and a sensitive substrate (e.g. a patterned surface, fiber tip, interference, hotspots) to further amplify the detection.

In the simplest detection scheme, the signal revealing the presence of the analyte is likely to be a measurable change of a spectral feature (e.g. overall intensity, a peak, a dip, an edge) of a reflected light or traveling light. Glass microrods, doped with metal NP, were proposed as anisotropic substrates for SERS detection of DNA strands [73]. As probes, the authors used molecular beacons, DNA hairpins labelled with a quenched fluorophore, whose emission is restored upon target binding and hairpin opening. Because the fluorophore also acted as Raman tag and binding increased its distance from the surface, SERS signal decreased in parallel to fluorescence increase, improving signal quality.

As of today, a number of commercial apparatus exists to detect spectral changes due to plasmon resonance. Evanescent waves platform readers are commercially available, where plasmonic signal can be detected on a multiwell geometry. In this fashion nanoparticles can be used not only as amplification for a targeted analyte but also as tracers; this is the case of [74] where signal of AuNP, as measured by a commercial platform reader (Epic Benchtop by Corning), is used to trace the intake of cells growing on the surface of a commercial multiwell. Using SPR prisms is another simple way to read evanescent wave signals from custom functionalized surfaces. For example, AuNP were immobilized and functionalized with DNA probes to produce a LSPR signal upon target binding (see Figure 3G), providing a multiplexed detection of sequences related to fungal pathogens [53]. In a similar setup, an impressive LOD of 100 aM was achieved for a panel of miRNA in real plasma samples from cancer patients [75]. Using colloid-based nanolithography, in [76] the authors fabricated a large-area nanoplasmonic sensor consisting of pairs of gold nanodisks which act as nanoantennas for miRNA detection. Springer et al. used AuNP and a surface functionalized with Ab to make a sandwich assay against carcinoembryonic antigen [77]. In the same style but with a strand displacement process miRNA can be detected using AuNP [78].

In this framework, optical fibers represent a cheap and reliable way to measure spectral changes. Even more so, optical fibers, providing an easy way to transport and manipulate light, tackle directly the problem of optical alignment, which is a potential weakness for optical biosensors, especially when the device is designed for POC or non-specialized lab environments.

With respect to the location of the sensitive part along the fiber, we can divide fiber-based biosensing techniques in two classes: (i) sensitive fiber tip; (ii) sensitive core/cladding structures.

#### 4.1.1 Sensitive fiber tip

The first class of techniques takes advantage of the combination of the microscopic reflective surface of the fiber tip (1–5 μm) and plasmonic structures fabricated by NP adhesion. This class of devices provides an enhanced change of reflection spectrum upon target binding. An advantage of such detection scheme is that the fiber can be inserted and moved around the sample to overcome transport limitation and it can be used to measure different samples in a multiwell format. An example of this class of systems was produced by coating the fiber facet with AuNP [79]. This device, based on LED illumination and spectrally-resolved detection by a miniature spectrometer, has been proposed to measure copper ions in water down to pM concentrations, although the same concept can be extended to biomolecular detection through immobilization of specific probes. Indeed, with gold triangular nanoprisms, selective detection with sub-fM LOD and extended linear range were achieved in a panel of miRNAs in serum [80, 81]. Further signal enhancement can be obtained through DNA-based strategies: metal nanostars tethered on the fiber tip were functionalized with so-called inverse SERS sentinels [82]. Upon binding of the target miRNA, the DNA probes fold into hairpins, bringing a fluorescent reporter closer to the surface and thus enhancing the Raman signal. The LOD for miRNAs in plant extracts was in the nM range.

**Figure 4:**
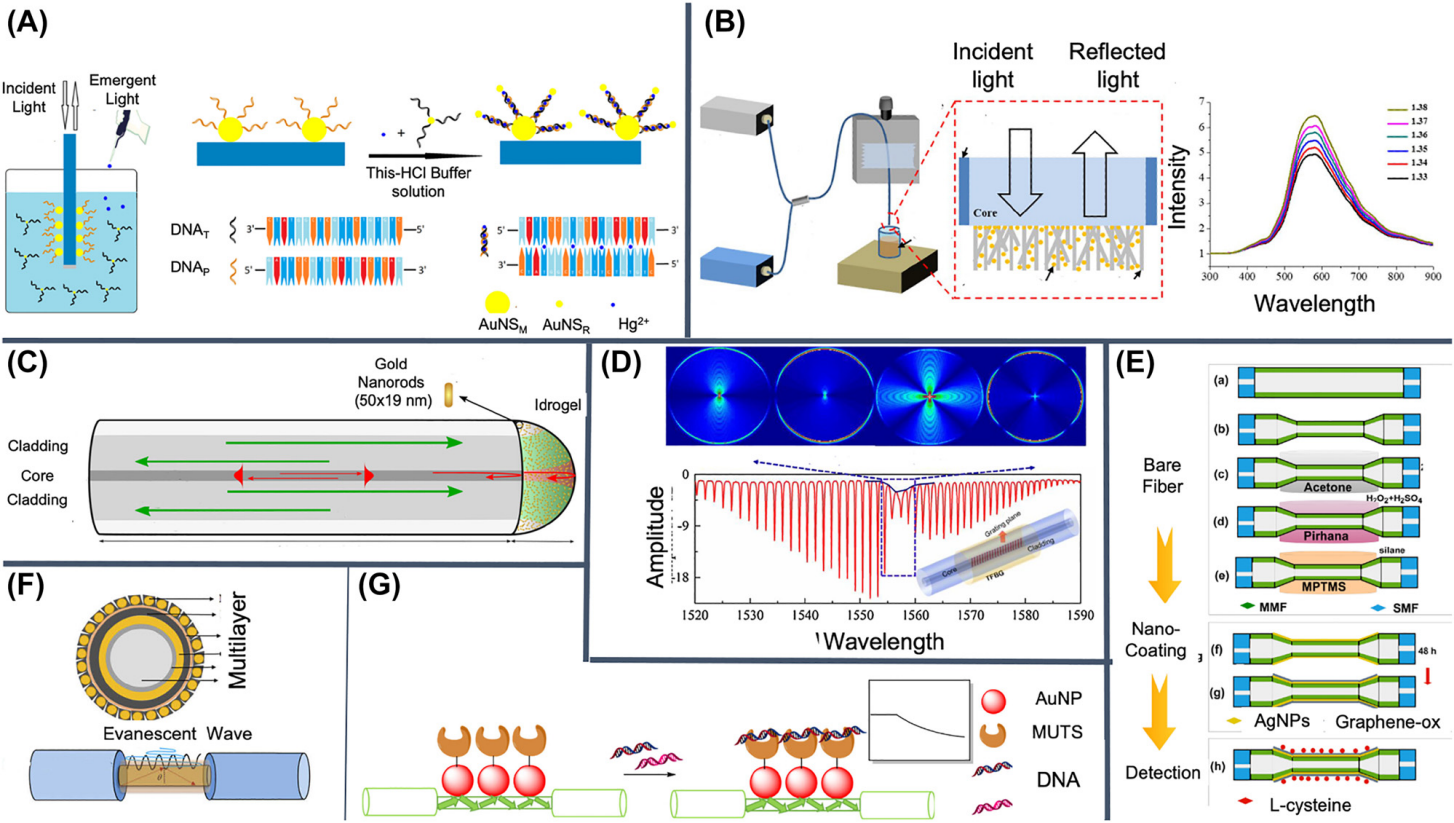
Fiber-based and spectrum resolved nanoparticle-assisted label-free biosensors. NP can be used to create sensitive 3D plasmonic nanostructures on the fiber tip by means of (A) DNA, (B) ZnO nanowires, or (C) a hydrogel providing also interferometric optical signal. Optical transported modes are sensitive to core/cladding structures decorated with NP like (D) fiber gratings, (E) tapered fibers, (F) multi-layer structures, and (G) functionalized AuNP. Reproduced with permission from: [83] (A), [85] (B), [86] (C), [87] (D), [88] (E), [89] (F), [90] (G).

AuNP can be self-assembled on the fiber tip by DNA-DNA interactions (Figure 4A), creating a fiber tip decorated with NP whose reflection spectrum depends on the analyte binding on the tip. In general DNA-DNA interaction is not only an ideal building block to obtain 3D structures, but also possesses a binding affinity sensitive to the surrounding conditions (e.g. Hg^2+^ concentration [83]). The shape of the NP decorating the fiber tip can be engineered to maximize the sensitivity, as in [84] where NP are cube shaped and attached to the surface. In this paper, Kim et al. also notably investigated the impact of fiber type (multi-mode vs. single-mode) on fiber positioning, that is how transported light modes inside the fiber can be impacted by fiber manipulation (e.g. bending). Single-mode fibers possess higher sensitivity than multi-mode fibers in principle, but their spectrum response is much more dependent than multi-mode fibers on bending and fiber positioning. In general, the three-dimensionality of the structures decorated with NP is a key factor to enhance the sensitivity of a fiber tip, which is to enhance the spectral response to a binding event. ZnO nanowires can be used to create 3D structures decorated with NP [85] that were demonstrated for the detection of PSA in water (Figure 4B).

Following this framework, a better sensitivity and richer signal can be obtained from a hydrogel on the tip of a fiber [86]. In this case, the plasmonic signal is not only enhanced by the larger 3D sensitive area, but also by the thickness of the uniform hydrogel structure, which creates a very sensitive Fabry–Perot interferometric spectrum on the fiber tip (Figure 4C).

**Figure 5:**
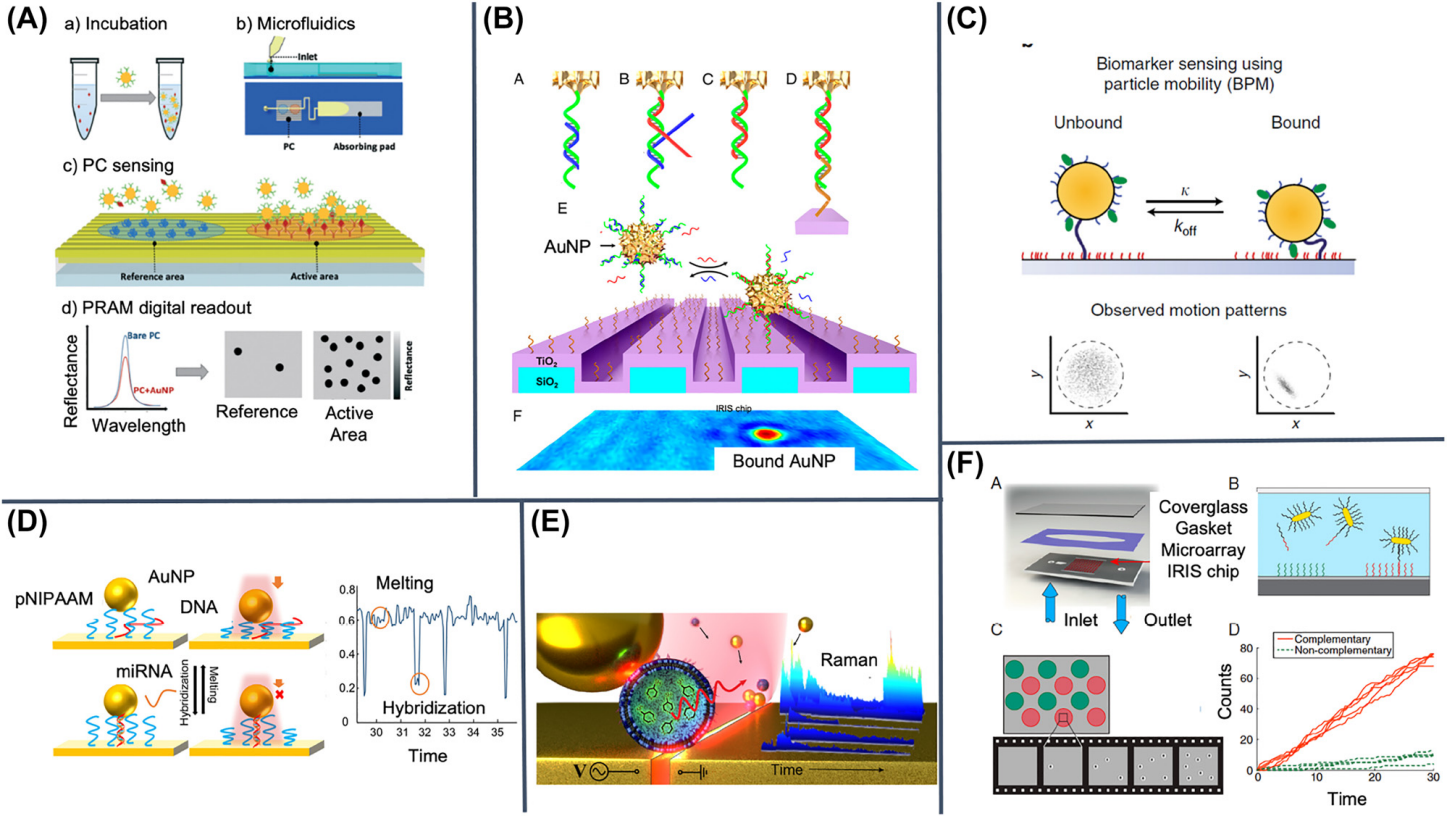
Spatially resolved nanoparticle-assisted label-free biosensors. Plasmonic NP interact with a metal-patterned plasmonic surface in presence of the analyte in (A) multi-spot microfluidic biosensor or (B) by a strand displacement detection process. To simplify the detection, NP can be linked to a surface and their mobility with and without the analyte can be used as detector: (C) NP tethered on a surface by a dsDNA; (D) NP immobilized over a temperature sensitive pNIPAAm layer. (E) NP can be used to amplify the signal once the analyte is captured by dielectrophoretic forces. (F) IRIS technique to count associating NP to reveal the presence of the analyte. Reproduced with permission from: [91] (A), [43] (B), [44] (C), [92] (D), [93] (E), [94] (F).

#### 4.1.2 Sensitive fiber core/cladding structures

A second large set of fiber-based biosensors relying on NP-assisted fabrication uses the central part of a fiber as a sensitive area, modifying the cladding-core structure so that the resulting intensity of the propagating optical modes become very dependent to the presence of the analytes. For the nature and the geometry of these technologies, they are often used in conjugation with a microfluidic part, so that the sensitive part is immersed in a microchannel or a microchamber, where samples can be moved in and out by means of micropumps or syringes.

In general optical transported modes are always dependent on evanescent wave effects involving the cladding. Huong et al. removed part of the cladding of a multi-mode fiber and modified the surface of the core using AuNP. Using a microfluidic geometry and by monitoring the spectrum at 522 nm, they were able to measure the presence of BSA with a LOD of 0.18 ng/mL (2 pM) [95]. A similar approach can be used to sense DNA mismatch using AuNP functionalized with MUTS protein, a mismatch DNA-repair protein [90] (Figure 4G). AuNP can be directly immobilized as a monolayer on the naked fiber core using thiols. By functionalizing the NP with ssDNA or ssRNA, nucleic acids in solution can be quantified with a LOD of 100 pM [96]. Sensitive structures can be also obtained using multi-layer structures on the fiber core. For example Liu et al. used a combination of polydopamine-MoSe2(Molybdenum diselenide) – AuNP-polydopamine to detect IgG in buffer solution with LOD of 0.054 μg/mL (0.36 nM) [89] (Figure 4F).

Agrawal et al. [88] used tapered multi-mode fibers to obtain a sensitive single mode part in the middle of the fiber, where silver nanoparticles (AgNP) and graphene oxide (GO) can be immobilized using self-assembly methods. The system enabled the detection of L-cystein in solution with LOD of 60 μM (Figure 4E).

Another valid way to make fiber-transported optical modes very sensitive to the presence of an analyte is to realize fiber gratings in the middle part of the fiber. In this way the sensitivity even to a small change of the refractive index to the surrounding liquid becomes detectable. Lee et al. use fiber grating and a scaffold of AuNP and polyacrylic coated with titanium-oxide to create a biosensor for creatinine [97].

Using fiber Bragg grating Wang et al. created a ”DNAzyme” biosensor for ultrasensitive Pb^2+^ detection on the basis of “hot spot” effect in near-infrared band. A AuNP-on-Au film construction was built by DNAzyme and its substrate strand. When Pb^2+^, with high catalytic activity, cleaves the DNAzyme-linked adenosine transforming DNA double helix into a ssDNA, the AuNP is free to interact with the sensor surface triggering a measurable detection event (LOD 8.56 pM) [87] (Figure 4D).

### 4.2 Spatially resolved NP-assisted detection

The large signal originating from a single NP detected by image-based label-free biosensors offers the capability of spatially resolved detection events. Among the advantages of this class of methods we can cite (i) the possibility to increase the number of events per unit time that are unlikely to be spatially superimposed on biosensor sensitive surface, and (ii) the possibility to perform multiplex experiments, hence enabling a fingerprinting of the analyte against different specific or non-specific probes. It is then a feature of this class of biosensors to bring the LOD down to the detection of a single binding event, generally to obtain a ”digital” biosensor (as in the case of single bio-nanoparticle detection in Section 5). A class of techniques achieving spatially resolved single particle detection relies on LSPR. Patterned structures on the surface covered with a metal oxide are a common choice to create plasmonic hotspots. Che et al. [91] used a UV-cured polymer as scaffold coated with TiO_2_ (Figure 5A) illuminated by a broadband light source, obtaining a narrow-band, nearly 100% reflective surface functionalized with antibodies. A sandwich assay with AuNP functionalized with the same antibodies resulted in a digital count of single molecule interaction events on the reflective image. In a low concentration regime, the volume-to-surface ratio is critical to obtain a low LOD, it is then not uncommon to integrate the detection scheme in a microfluidic circuit. In the case of [91], the microfluidic chip also contained more than one sensitive spot, so to have a multiplexed measurement.

Si0_2_ scaffolds, based on MOS (metal-oxide-semiconductor) technology, offer a high geometrical quality for creating plasmonic hotspots. It is the case of the technique in [43], where, by reflective imaging, it was possible to localize and count the interactions between AuNP and a TiO_2_-coated Si surface. This particular device (Figure 5B) is also an example of a detection scheme based on DNA strand displacement: such method minimizes the influence of non-specific interactions between the NP and the surface by using a “protector” DNA strand, which is displaced only in presence of the analyte.

Selective reflection imaging to localize the NP typically requires a calibrated, wavelength-specific setup. In contrast, dark-field imaging can be sensitive to the presence of a single NP, well below the diffraction limit, as long as the stray light and the signal-to-noise ratio are compatible with the detection. One way to enhance the detection signal is to impose controlled boundary conditions to the NP. Visser et al. [44] used a dsDNA to link AuNP to a biosensor surface scanned by dark field imaging; dsDNA lengths were chosen so to provide an optimal mobility (Figure 5C). Both the NP and the surface were functionalized with antibodies or DNA or aptamers. Tethered NP were coated with capture strands with high affinity for the target, while the substrate was functionalized with shorter, lower affinity detection strands, so that in the presence of the analyte the NP forms a sandwich assay on the surface. Such change in the particle mobility, corresponding to NP bound states, is recorded by particle tracking in time. In this way, not only single-molecule sensitivity is achieved, but also the kinetic of association and dissociation is accessible in the experiments. Chen et al. [92] forced a vertical oscillation of AuNP using a temperature sensitive polymer (pNIPAAm) periodically heated by a laser. When a miRNA binds to a ssDNA attached to the surface, it creates a rigid structure that modifies the oscillating motion of the AuNP, thus revealing the presence of miRNA (Figure 5D). In this work, an imaging system was also implemented to monitor scattering and SPR imaging at the same time, thus counting and localizing the binding–unbinding events.

Analytes can often be identified by spectroscopic fingerprinting, such as those based on Raman spectroscopy. However, such methods are typically not suitable for spatially resolved detection, because the analytes can be sparse on the surface and the signal is low. This issue was addressed by Ertsgaard et al. [93] using dielectrophoretic forces produced by a patterned gold surface to capture nanovesicles on the sharp edges of the electrodes. AuNP are then captured by the trapped nanovesicle and worked as nanoantennas for Raman signal; using a line-shaped spectrum-controlled light it was then possible to localize NP on the electrode (Figure 5E).

The use of patterned surfaces can enhance the plasmonic signal, but it can also pose a problem of fragility and quality of the biosensor chips. Biosensors based on light interference can avoid such problems. In the case of NP, the interference takes place between the planar electromagnetic field *E*_r_ reflected by the surface and the spherical wave *E*_s_ scattered by a NP binding on it. In principle, the intensity interference term (∝*E*_s_ ⋅ *E*_r_) not only scales with the cube of the NP radius [98], but also depends on the stable and controllable *E*_r_ to amplify the smaller field produced by the NP. One way to tailor *E*_r_ is to exploit thin layer interference from a surface coating. Sevenler et al. used a silicon coated surface chip where the interference between the reflected image and the AuNP can be monitored using a 20x objective scanning at various focus distances, either on a microplate [99] or in a microfluidic environment [94] (Figure 5F). The digital counting of natural NP, and their association and dissociation events without the need of other NP for signal amplification, is discussed in Section 5.

## 5 Digital detection of viral particles

The key challenge in imaging nanoscale objects on a surface arises from the rapid drop in the scattering cross-section of the object as it becomes much smaller than the illumination wavelength *λ*. This challenge can generally be declined into three main parameters: resolution, specificity and sensitivity [100]. Spatial resolution, the minimum distance at which two distinct points in a specimen can be distinguished, is ultimately limited by diffraction. The image of an object much smaller than the wavelength, the so-called point-spread function (PSF), has a finite size (∼*λ*/*NA* and ∼*λ*/*NA*^2^ in the transverse and in the axial direction, respectively), which primarily depends on the wavelength *λ* of the light and on the numerical aperture *NA* of the objective. The exact shape of the PSF depends on the details of the illumination and the collection optics. Importantly, if the surface density of the detected particles is low enough (*i.e.* if their mutual distance is sufficiently large) and in the presence of an accurately characterized PSF, the three-dimensional position of the center of mass of each particle can be determined with a precision well below the size of the PSF, by fitting the PSF with a suitable model function [101]. In the context of microscopy, specificity refers to the ability to distinguish signals from different types of objects of interest within a sample. This can be achieved by a variety of strategies: quantification of the amplitude of a detected signal (to estimate mass or optical contrast of the particles), surface functionalization with suitable receptors to promote specific binding, and the use of different fluorescence or scattering labels. Finally, sensitivity is the ability to discriminate the signal from an object of interest from any form of background noise (e.g. dark noise, shot noise, intensity fluctuations associated to a non-static environment) [102].

### 5.1 Scattering-based imaging of single viruses

**Figure 6:**
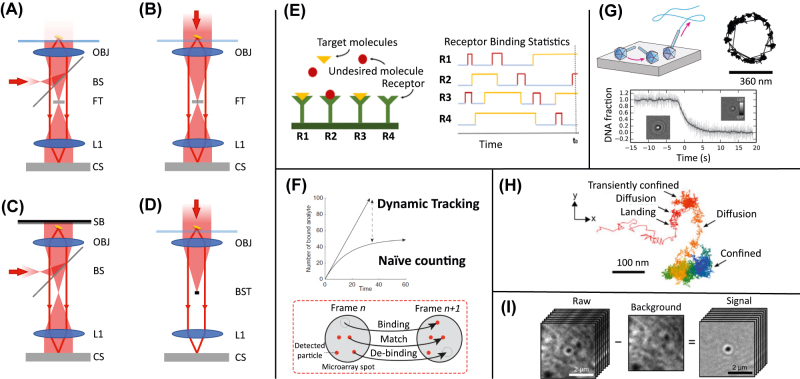
Digital detection of bio-nanoparticles. (A)–(D) Simplified schematic representation of common microscopy configurations enabling label-free imaging of single bio-nanoparticles: (A) interferometric scattering (iSCAT), (B) coherent bright-field (COBRI), (C) single-particle interferometric reflectance imaging sensor (sp-IRIS), and (D) dark field, respectively (SB = substrate, OBJ = objective lens, BS = beam splitter, FT = filter, BST = beam stop, L1 = relay lens, CS = camera sensor). In all cases, shaded red areas represent the illumination light path, while continuous red lines correspond to the image-forming light path. (E) Pictorial representation of the theoretical approach proposed in Ref. [105] which, based on the statistical analysis of the kinetics of single binding events, enables detecting high-affinity, ultra-diluted targets in the presence of non-specific competitors with improved sensitivity. (F) Schematic representation of time- and space-resolved analysis of binding events proposed in Ref. [94], enabling to overcome the limitations of end-point methods in the detection of ultra-diluted targets. (G) iSCAT microscopy-based tracking of a single bacteriophage tethered on an APTES-functionalized glass surface. In the right panel a portion of circular trajectory performed by the head of the phage is shown. The lower panel illustrates the ability of iSCAT microscopy to provide a quantitative, time-resolved measurement of the mass of an immobilized particle: the observed change in the optical contrast of the phage’s image corresponds to the ejection of DNA upon interaction with the receptor protein LamB, as pictorially shown in the upper left panel. (H) 2D projection of the trajectory corresponding to the localized motion of a vaccinia virus particle landing on the membrane of an epithelial cell, as obtained from COBRI microscopy. High-resolution tracking of single virus particles in such a complex environment relies on the possibility of effectively removing any background contribution, as shown in (I), where the background intensity distribution, associated with the slowly-fluctuating environment, is estimated as the time average of the collected image sequence. Reproduced with permission from: [105] (E), [94] (F), [106] (G), [107] (H, I).

A conceptually simple strategy to achieve high sensitivity in the detection of single bio-nanoparticles consists in the highly selective collection of the light scattered by the particle alone, while rejecting all other contributions. Experimentally, this so-called *dark-field* condition can be obtained for example by selectively illuminating the sample with an evanescent wave (total internal reflection scattering microscopy [103]), by using a strongly tilted illumination (possibly in combination with a reflecting surface placed behind the sample, as proposed in Ref. [104]), or by blocking the light transmitted by the sample *via* a suitably placed beam stop, as schematically shown in Figure 6D.

An alternative strategy consists in adopting combinations of sample illumination and collection of scattered light, such that, in the collected images, the optical contrast is provided by the interference of the light scattered by the particles and a suitable reference wave, with optimized wavelength, propagation direction, amplitude, and relative phase. Common configurations exploiting this principle are iSCAT, COBRI and spIRIS. iSCAT (interferometric scattering) usually relies on the interference between the light back-scattered by the sample and the light reflected from an interface close to the sample, typically a coverslip [108], as shown in Figure 6A. Recently, iSCAT has been used to track with millisecond temporal resolution and nanometer spatial resolution the rototranslational dynamics of bacteriophages tethered on a silanized glass surface [106]. Moreover, quantitative, time-resolved measurement of the optical contrast of single phages immobilized on the surface was used to monitor the process of DNA ejection from the phage’s head triggered by the interaction with the receptor protein LamB (see Figure 6G). Coherent brightfield (COBRI) microscopy can be considered the transmission counterpart of iSCAT (see Figure 6B). Single-particle Interferometric reflectance imaging sensor (sp-IRIS) is an experimental technique combining interferometric imaging principles with a specialized substrate, fabricated from a silicon wafer covered by a layer of silicon dioxide [109] (see Figure 6C). Besides providing a reference reflected wave with optimized amplitude and phase, the substrate can be coated with antibodies or other receptors to facilitate selective binding of a species of interest. For example, in Ref. [45] sp-IRIS was demonstrated for the multiplexed detection of three different recombinant vesicular stomatitis viruses. Viral particles are incubated for 10–60 min with DNA-conjugated antibodies targeting specific proteins of each virus type. The solution is then put in contact with a DNA microarray prepared on the IRIS chip for detection.

Relying on the interference of the scattered light with a reference beam, all the cited microscopy configurations (iSCAT, COBRI and sp-IRIS) can be classified as heterodyne methods, characterized by a *linear* relation between the scattered electric field and the detected intensity. A key consequence of this linearity, where contributions from different scatterers simply add up in the image, is that heterodyne methods offer the possibility of removing stray-light or static or quasi-static background contribution. For example, in Ref. [107] COBRI microscopy is used to track with high spatial and temporal resolution a single viral particle landing and interacting with the plasma membrane of a live epithelial cell (Figure 6H). The marked time-scale separation between the rapid motion of the virions and the relatively slow dynamics of the cell enables estimating the (rather strong) scattering background from the cell as the average (over a suitable time window) of the collected images. Subtraction of the so-estimated background from the original images leads to high-contrast images of the virion alone (see Figure 6I). Notably, this procedure would not be possible for homodyne detection, as in the case of dark field imaging with coherent (laser) illumination. In this case, the light scattered from different objects sums up on a coherent basis and what is observed in the image is the interference pattern generated by the different contributions. As a consequence, dark-field microscopy is suitable for the investigation of very clean samples [110], while *in-vivo* studies and detection in complex matrices are much more challenging.

### 5.2 Exploiting particles dynamics

The rich, space- and time-resolved information provided by imaging-based platforms opens to a number of exciting possibilities, which are not available for end-point or space-averaging methods. A representative example along this line, which appears to be very promising in boosting the sensitivity of biosensors with time-resolved single-particle capability is the one described in Ref. [94]. Single binding/unbinding events of the targets are accurately monitored over time. Instead of counting over time the instantaneous number of bound targets (naïve counting), the authors considered the cumulative number of binding events (dynamic tracking). The second approach is shown to outperform the first one, in particular in conditions of ultralow analyte concentrations, where the equilibrium number of bound targets is very low (Figure 6F). The method is demonstrated using DNA-decorated gold nanorods, which are detected on a complementary DNA-coated sp-IRIS chip.

Another example of how the accurate characterization of the spatiotemporal dynamics of the targets particles can improve the selectivity of the detection process is provided by the previously mentioned (Section 2.1) Ref. [15]. Here, the measurement of the mobility of the particles detected close to a surface coated with specific capture molecules is shown to enable highly selective recognition against non-target virions.

On the other hand, if the particle is detected while in solution, the quantitative measurement of its mobility (*i.e.* of its diffusion coefficient) can provide, via a suitable Stokes–Einstein relation, information on its size, even if it is well below the resolution limit of the imaging system [110, 111]. Another very common and general limitation in the detection of ultra-diluted targets is the simultaneous presence in solution of other competing molecules/particles which could also bind to the receptors. Even if the affinity of the competitors to the receptors is low, if they are abundant, the non-specific signal associated with their binding to the receptor could be substantial and lead to a severe overestimation of the target concentration. In the absence of a complementary mechanism enabling to discriminate the nature of the target (based for example on shape, mass or mobility), end-point methods can be heavily affected by this effect. In Ref. [105] a simple and general method to distinguish between specific and non-specific binding is proposed, based on a statistical analysis of single binding events. The proposed scheme, named dynamic tracking (DT) biosensing, relies on the identification of a threshold time separating short-lasting (likely non-specific) binding events and long-lasting (likely specific) binding events, as schematically shown in Figure 6E. Also with the support of numerical simulations, the authors show that an optimized DT scheme can reach a sensitivity up to 4 orders of magnitude higher than an end-point sensor, for given receptors and sample composition.

## 6 Single molecule detection

Biosensors with single particle detection capability were already mentioned in Section 5. Here we present a restricted class of devices with an extremely high sensitivity enabling the direct detection of single molecules, hence with sizes much smaller than bio-nanoparticles, and without employing extrinsic NP to amplify the signal (Section 4). When analytes are individual molecules much smaller than the wavelength of light, the optical signal is ascribed to the light scattered by a single oscillating dipole. The main challenge in this case is to obtain a signal that depends mostly on the light interacting with the target molecular dipole and not from environmental noise or from other molecules. This can be achieved by creating a very small “sensitive volume”, in principle with a size comparable to that of the target molecule, specifically captured by a bio-recognition element. Evanescent waves are an example of such paradigm, where the illuminated volume is reduced to the decay length of an electromagnetic wave at the interface between two materials. This concept can be suitable for single NP detection, but it is not enough to obtain a robust signal from single molecules, so other solutions must be employed, such as additional mechanisms to amplify the detection. Resonant structures, either dielectric or plasmonic, and localized plasmonic effects are ideal environments to achieve single-molecule sensitivity.

**Figure 7:**
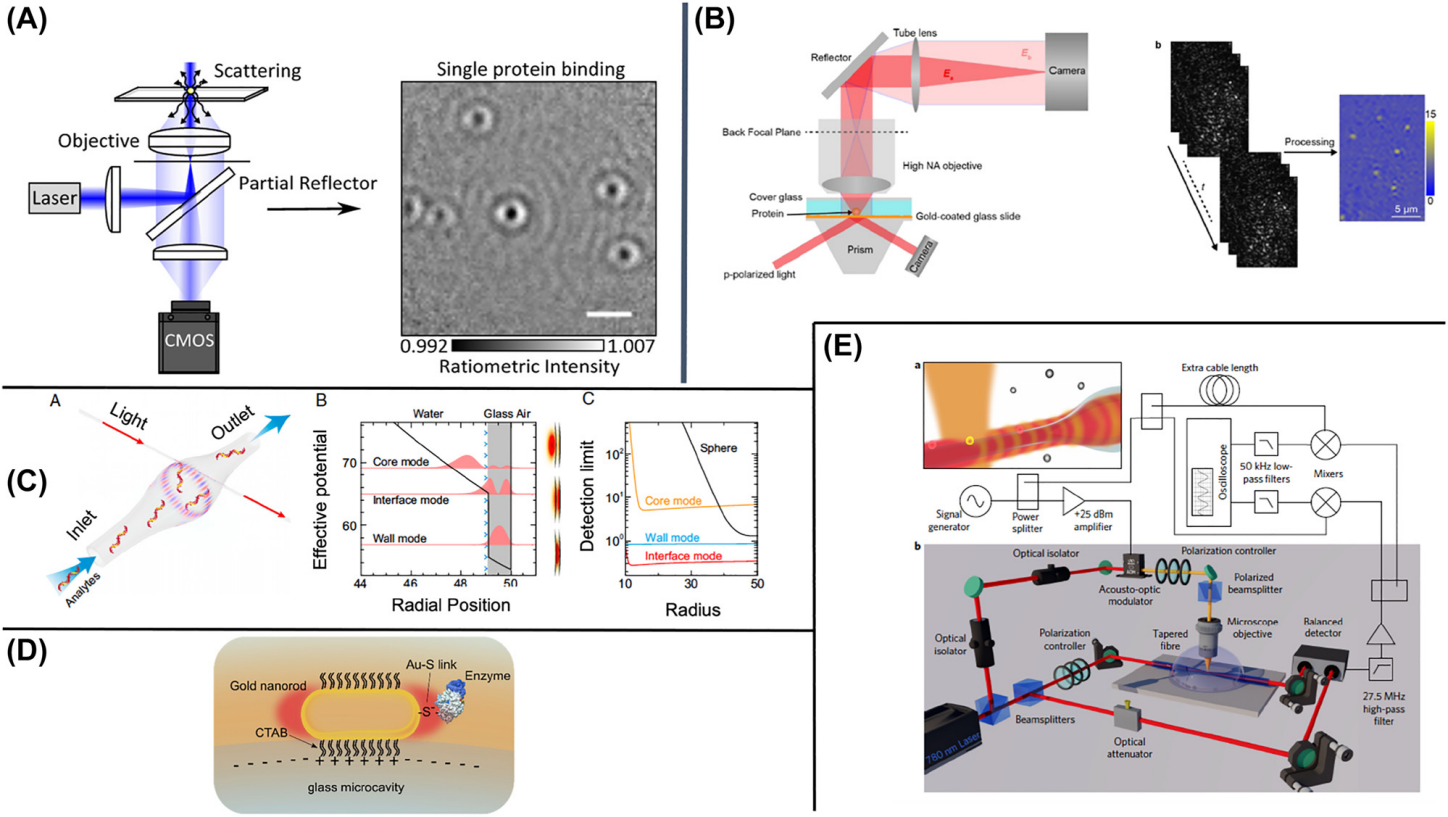
Single molecule detection biosensors. (A) Scattering interference equalized using a spatially modulated reflector so to attenuate specifically the reflected light. (B) Simultaneous detection of TIR and LSPR signal. (C) Whispering gallery modes in a capillary microbubble enhanced by Au nanorods. (D) Glass bead resonator enhanced by Au nanords. (E) Optical fibers are photonic structures that become ultra-sensitive when tapered into micro-fibers (nano-fibers). Reproduced with permission from: [112] (A), [113] (B), [114] (C), [115] (D), [116] (E).

Cole et al. [112] introduced a partial reflector, that is a spatially modulated reflector so to attenuate specifically the reflected light, on a scattering-interference (iSCAT) scheme (see paragraph 5 for detail on iSCAT) to optimize the interference, as shown in Figure 7A. Using the coherent light of a scanning laser to obtain a high quality interferometric image and a combination of frame averages and frame ratios, it is possible to reveal single proteins captured on the biosensor surface.

The interference between a plasmonic surface resonance, excited by an evanescent wave, and a single molecule scatterer can be optimized to detect single proteins or antibodies [113, 117] using the optical scheme in Figure 7B. Notably, in this optical setup the SPR signal and total internal reflection signal can be recorded simultaneously, thus enriching the measured binding events and offering enhanced control on the molecular behaviour.

Optical or plasmonic resonances are in principle extremely sensitive signals for single molecule biosensors, especially because the resonant state can amplify the single molecule binding signal above the background. The challenge to create a resonator small enough in size and sharp enough in the spectral response to be sensitive to a single molecule has been addressed in the last years by a variety of techniques based on the combination of optical resonance modes (e.g. photonic rings, waveguides, WGM) and plasmonic resonances (e.g. AuNP, metal tips, nanocavities [118]).

WGM sensors [119] exploit the change of optical resonance frequency to detect the analytes. The optical resonance is excited by coupling light into a circular photonic structure, which creates a stable resonant spectrum sensitive to the presence of analytes. Furthermore, to amplify the presence of the target molecule, a plasmonic particle can be used. Yu et al. [114] exploited the interaction between optical modes of a microbubble cavity fabricated in glass capillary and immobilized gold nanorods to create an optofluidic biosensor (Figure 7C). Using a laser to scan the optical response while the sample is flowing inside the nanorods-decorated nanocavity, they were able to use the so-called “interface modes” as a transducer to detect single DNA strands. With a similar approach, Subramanian et al. [115, 120] exploited the optical resonant modes in a microsphere enhanced by an Au nanorods immobilized on its surface to detect single sub-kDA molecules. Remarkably, this approach also enabled sensing the conformational changes of an enzyme (Figure 7D).

Another class of single-event biosensors of growing importance is based on nano-fibers. An optical fiber is extremely tapered so to create a tiny sensitive area; in this way the optical transport properties of the fiber become very dependent on the presence of molecules attached to the fiber surface. Remarkably, using this approach combined with the optical scheme in Figure 7E, quantum-limited precision biosensor can be obtained [116]. In general such a tiny fiber works as a highly sensitive intermodal interferometer and generates interference fringes in the transmission spectrum. To extremely amplify this effect, the surface of the tapered fiber can be also decorated with plasmonic nanostructures [121], obtaining a remarkable sub-aM LOD for ErB2 growth factor receptor in buffer.

## 7 Conclusions and outlook

The selection included in this review is far from being exhaustive of the rapidly expanding field of optical-label free biosensors. We focused on specific topics addressed in Sections 2–6, and among these we selected only a portion of the numerous studies published in the last five years. Despite the limited sampling of the scientific literature in this field, the large variety of features and performances of the addressed detection methods is remarkable. As shown in Figure 1, the LOD seems rather homogeneously distributed across as much as 15 orders of magnitude of target concentrations. It must be pointed out that the LOD, *i.e.* the minimum concentration of target that can be reliably detected, is not necessarily related to the sensitivity of the detection method, which represents the response of the optical signal upon adhesion of a unit amount of substance on the sensing surface. In Figure 1, we chose to report the LOD and not the sensitivity, because the former is directly related to the application of the biosensor, whereas the latter is rarely reported and generally difficult to compare between different techniques and targets. For optical label-free biosensors, the sensitivity is ultimately given by the lowest value of refractive index change that induces a detectable signal above the background noise. For methods of the same class and for the same type of target, similar sensitivities are often reported, or at most within one or two order of magnitude. As discussed in Section 6, a few exceptional technical solutions have been proposed to dramatically increase the sensitivity down to single molecule detection. However, the LOD of a method, especially when employed with a real matrix, depends on many factors in addition to the sensitivity, including specificity of binding, passivation to non-specific adsorption, amount and localization of the surface probes relative to the volume of the target solution, and transport effects. Moreover, detection methods are often designed for specific classes of targets, which present their own typical concentrations in real conditions, as in the case of diagnostic applications (Section 2), practically spanning the entire range of LOD of Figure 1. These arguments may explain why the methods addressed in this review, and optical label-free methods in general, cover so many orders of magnitudes of LOD, when expressed in molar concentration. However, Figure 1 also shows that the LOD of the studies addressing bio-nanoparticles (green segments) are typically very low, whereas those addressing small molecules (blue segments) are on the large values side of the LOD scale. This is due to the fact that optical label-free methods are basically sensitive to the polarizability of the target analyte, which scales with its volume and refractive index. With the exception of the methods relying on NP-assisted detection (Section 4), all biological targets fall within a narrow range of refractive indexes and densities [122], hence the field amplitude per single target, from small molecules up to bio-nanoparticles, substantially scales with the volume of the target itself. This makes optical label-free biosensors particularly sensitive to bio-nanoparticles down to digital detection of single particles, as discussed in Section 5, or suitable to be combined with mass amplification mechanisms designed by DNA nanotechnology tools, as described in Section 3. Another distinctive feature of label-free biosensors is the capability of measuring the binding in real-time, hence giving access to the interaction kinetics. However, as displayed in Figure 1 (dashed line segments), this capability has been exploited only by a fraction (about a third) of the methods addressed in this review, rather uniformly across the LOD scale and target types. Further improvements are expected by implementing some form of kinetic analysis in all methods, to better discriminate between specific and non-specific binding or to optimize the time-to-result through real-time adaptive monitoring of the binding process.

All these distinctive features of optical label-free biosensors represent potential competitive advantages and can arguably drive the success of their future commercial exploitation. In the case of diagnostic applications, the dramatic lack of novel, high performance biosensors among the systems exploited in the fight to the Covid-19 pandemic suggests that some fundamental step is still critical between excellent results in lab conditions and widespread use in routine diagnostics. Bringing a novel diagnostic tool up to competitive levels of reproducibility, robustness and scalability of production require enormous investments, and long work typically outside the scope of academic research. On the contrary, standard methods such as LFD, ELISA and PCR have the advantage of being far ahead on these aspects and it is increasingly difficult to close the gap over time. Therefore, the introduction of a biosensor as widely used diagnostic tool must also offer a very significant and not marginal advantage in order to replace established methods recognized as standards. Some of the distinctive features of optical label-free biosensors emphasized in this review, when combined to a mature enough system, can provide such an advantage and make a difference in the competition for next generation diagnostic devices.

## References

[1] Schasfoort R. B. M. (2017). Handbook of Surface Plasmon Resonance.

[2] Altug H., Oh S. H., Maier S. A., Homola J. (2022). Advances and applications of nanophotonic biosensors. Nat. Nanotechnol..

[3] Mercer T. R., Salit M. (2021). Testing at scale during the COVID-19 pandemic. Nat. Rev. Genet..

[4] Vandenberg O., Martiny D., Rochas O., Alex van Belkum, Kozlakidis Z. (2021). Considerations for diagnostic COVID-19 tests. Nat. Rev. Microbiol..

[5] Falzone L., Gattuso G., Tsatsakis A., Spandidos D. A., Libra M. (2021). Current and innovative methods for the diagnosis of COVID-19 infection (Review). *Int. J. Mol. Med.*.

[6] Quammen D. (2012). *Spillover: Animal Infections and the Next Human Pandemic*.

[7] Zanchetta G., Lanfranco R., Giavazzi F., Bellini T., Buscaglia M. (2017). Emerging applications of label-free optical biosensors. Nanophotonics.

[8] Mauriz E., Dey P., Lechuga L. M. (2019). Advances in nanoplasmonic biosensors for clinical applications. Analyst.

[9] Ong D. S. Y., Fragkou P. C., Schweitzer V. A., Chemaly R. F., Moschopoulos C. D., Skevaki C. (2021). How to interpret and use COVID-19 serology and immunology tests. Clin. Microbiol. Infect..

[10] Soler M., Estevez M. C., Cardenosa-Rubio M., Astua A., Lechuga L. M. (2020). How nanophotonic label-free biosensors can contribute to rapid and massive diagnostics of respiratory virus infections: COVID-19 case. ACS Sens..

[11] Lee S. L., Kim J., Choi S., Han J., Seo G., Lee Y. W. (2021). Fiber-optic label-free biosensor for SARS-CoV-2 spike protein detection using biofunctionalized long-period fiber grating. Talanta.

[12] Kawasaki D., Yamada H., Sueyoshi K., Hisamoto H., Endo T. (2022). Imprinted photonic crystal-film-based smartphone-compatible label-free optical sensor for SARS-CoV-2 testing. Biosensors.

[13] Yano T. a., Taira K., Ono M. (2022). Ultrasensitive detection of SARS-CoV-2 nucleocapsid protein using large gold nanoparticle-enhanced surface plasmon resonance. Sci. Rep..

[14] Huang L., Ding L., Zhou J. (2021). One-step rapid quantification of SARS-CoV-2 virus particles via low-cost nanoplasmonic sensors in generic microplate reader and point-of-care device. Biosens. Bioelectron..

[15] Li N., Wang X., Joseph T. (2022). Label-free digital detection of intact virions by enhanced scattering microscopy. J. Am. Chem. Soc..

[16] Zheng Y., Bian S., Sun J., Wen L., Rong G., Sawan M. (2022). Label-free LSPR-vertical microcavity biosensor for on-site SARS-CoV-2 detection. Biosensors.

[17] Paria D., Kwok K. S., Raj P., Zheng P., Gracias D. H., Barman I. (2022). Label-free spectroscopic SARS-CoV-2 detection on versatile nanoimprinted substrates. *Nano Lett.*.

[18] Murillo A. M. M., Tomé-Amat J., Ramírez Y. (2021). Developing an Optical Interferometric Detection Method based biosensor for detecting specific SARS-CoV-2 immunoglobulins in serum and saliva, and their corresponding ELISA correlation. Sens. Actuators, B.

[19] Calvo-Lozano O., Sierra M., Soler M. (2022). Label-free plasmonic biosensor for rapid, quantitative, and highly sensitive COVID-19 serology: implementation and clinical validation. Anal. Chem..

[20] Qu J. H., Leirs K., Maes W. (2022). Innovative FO-SPR label-free strategy for detecting anti-RBD antibodies in COVID-19 patient serum and whole blood. ACS Sens..

[21] Adi W., Biswas D., Shelef M. A., Yesilkoy F. (2022). Multiplexed COVID-19 antibody quantification from human sera using label-free nanoplasmonic biosensors. Biomed. Opt. Express.

[22] Yue Y., Ding H., Chen C. (2021). Label-free optical antibody testing kit based on a self-assembled whispering-gallery-mode microsphere. J. Biophotonics.

[23] Loyez M., Lobry M., Hassan E. M., DeRosa M. C., Caucheteur C., Wattiez R. (2021). HER2 breast cancer biomarker detection using a sandwich optical fiber assay. Talanta.

[24] Sypabekova M., Amantayeva A., Vangelista L., González-Vila Á., Caucheteur C., Tosi D. (2022). Ultralow limit detection of soluble HER2 biomarker in serum with a fiber-optic ball-tip resonator assisted by a tilted FBG. *ACS Meas. Sci. Au*.

[25] Chiavaioli F., Zubiate P., Villar I. D. (2018). Femtomolar detection by nanocoated fiber label-free biosensors. ACS Sens..

[26] Esposito F., Sansone L., Taddei C., Campopiano S., Giordano M., Iadicicco A. (2018). Ultrasensitive biosensor based on long period grating coated with polycarbonate-graphene oxide multilayer. Sens. Actuators, B.

[27] Esposito F., Sansone L., Srivastava A. (2021). Long period grating in double cladding fiber coated with graphene oxide as high-performance optical platform for biosensing. Biosens. Bioelectron..

[28] Dey P., Fabri-Faja N., Calvo-Lozano O. (2019). Label-free bacteria quantification in blood plasma by a bioprinted microarray based interferometric point-of-care device. ACS Sens..

[29] Hendriks J., Stojanovic I., Schasfoort R. B. M., Saris D. B. F., Karperien M. (2018). Nanoparticle enhancement cascade for sensitive multiplex measurements of biomarkers in complex fluids with surface plasmon resonance imaging. Anal. Chem..

[30] Nguyen D. K., Jang C. H. A label-free liquid crystal biosensor based on specific dna aptamer probes for sensitive detection of amoxicillin antibiotic. Micromachines.

[31] Picciolini S., Gualerzi A., Vanna R. (2018). Detection and characterization of different brain-derived subpopulations of plasma exosomes by surface plasmon resonance imaging. Anal. Chem..

[32] Nguyen D. K., Jang C. H. A cationic surfactant-decorated liquid crystal-based aptasensor for label-free detection of malathion pesticides in environmental samples. Biosensors.

[33] Geng Z., Zhang X., Fan Z., Lv X., Su Y., Chen H. (2017). Recent progress in optical biosensors based on smartphone platforms. Sensors.

[34] Sanjay M., Singh N. K., Ngashangva L., Goswami P. (2020). A smartphone-based fiber-optic aptasensor for label-free detection of: Plasmodium falciparum glutamate dehydrogenase. Anal. Methods.

[35] Zhang J., Khan I., Zhang Q. (2018). Lipopolysaccharides detection on a grating-coupled surface plasmon resonance smartphone biosensor. Biosens. Bioelectron..

[36] Yesilkoy F., Terborg R. A., Pello J. (2018). Phase-sensitive plasmonic biosensor using a portable and large field-of-view interferometric microarray imager. Light Sci. Appl..

[37] Miyazaki C. M., Kinahan D. J., Mishra R. (2018). Label-free, spatially multiplexed SPR detection of immunoassays on a highly integrated centrifugal lab-on-a-disc platform. Biosens. Bioelectron..

[38] Lai M., Slaughter G. (2019). Label-free microRNA optical biosensors. Nanomaterials.

[39] Xiao M., Lai W., Man T. (2019). Rationally engineered nucleic acid architectures for biosensing applications. Chem. Rev..

[40] Huertas C. S., Calvo-Lozano O., Mitchell A., Lechuga L. M. (2019). Advanced evanescent-wave optical biosensors for the detection of nucleic acids: an analytic perspective. *Front. Chem.*.

[41] D’Agata R., Spoto G. (2019). Advanced methods for microRNA biosensing: a problem-solving perspective. Anal. Bioanal. Chem..

[42] Dass M., Gür F. N., Kołataj K., Urban M. J., Liedl T. (2021). DNA origami-enabled plasmonic sensing. J. Phys. Chem. C.

[43] Canady T. D., Li N., Smith L. D. (2019). Digital-resolution detection of microRNA with singlebase selectivity by photonic resonator absorption microscopy. Proc. Natl. Acad. Sci. U. S. A..

[44] Visser E. W. A., Yan J., Van IJzendoorn L. J., Prins M. W. J. (2018). Continuous biomarker monitoring by particle mobility sensing with single molecule resolution. Nat. Commun..

[45] Seymour E., Ünlü N. L., Carter E. P., Connor J. H., Ünlü M. S. (2021). Configurable digital virus counter on robust universal DNA chips. ACS Sens..

[46] Daems D., Pfeifer W., Rutten I., Saccà B., Spasic D., Lammertyn J. (2018). Three-dimensional DNA origami as programmable anchoring points for bioreceptors in fiber optic surface plasmon resonance biosensing. ACS Appl. Mater. Interfaces.

[47] Daems D., Rutten I., Bath J. (2019). Controlling the bioreceptor spatial distribution at the nanoscale for single molecule counting in microwell arrays. ACS Sens..

[48] He C., Wang M., Sun X. (2019). Integrating PDA microtube waveguide system with heterogeneous CHA amplification strategy towards superior sensitive detection of miRNA. Biosens. Bioelectron..

[49] Janik M., Hamidi S. V., Koba M. (2021). Real-time isothermal DNA amplification monitoring in picoliter volumes using an optical fiber sensor. Lab Chip.

[50] Huertas C. S., Bonnal S., Soler M., Escuela A. M., Valcárcel J., Lechuga L. M. (2019). Site-specific mRNA cleavage for selective and quantitative profiling of alternative splicing with label-free optical biosensors. Anal. Chem..

[51] Khateb H., Klös G., Meyer R. L., Sutherland D. S. (2020). Development of a label-free LSPR-apta sensor for Staphylococcus aureus detection. ACS Appl. Bio Mater..

[52] Lynn N. S., Špringer T., Slabý J. (2019). Analyte transport to micro-and nano-plasmonic structures. Lab Chip.

[53] Zopf D., Pittner A., Dathe A. (2019). Plasmonic nanosensor array for multiplexed dna-based pathogen detection. ACS Sens..

[54] Hübner K., Pilo-Pais M., Selbach F. (2019). Directing single-molecule emission with DNA origami-assembled optical antennas. Nano Lett..

[55] Trofymchuk K., Glembockyte V., Grabenhorst L. (2021). Addressable nanoantennas with cleared hotspots for single-molecule detection on a portable smartphone microscope. Nat. Commun..

[56] Barrows J. K., Van Dyke M. W. (2022). Biolayer interferometry for DNA-protein interactions. PLoS One.

[57] Carzaniga T., Zanchetta G., Frezza E. (2021). A bit stickier, a bit slower, a lot stiffer: specific vs. nonspecific binding of gal4 to dna. Int. J. Mol. Sci..

[58] Townshend B., Xiang J. S., Manzanarez G., Hayden E. J., Smolke C. D. (2021). A multiplexed, automated evolution pipeline enables scalable discovery and characterization of biosensors. Nat. Commun..

[59] Muhammad M., Shao C. S., Huang Q. (2021). Aptamer-functionalized Au nanoparticles array as the effective SERS biosensor for label-free detection of interleukin-6 in serum. *Sens. Actuators, B*.

[60] Villa A., Brunialti E., Dellavedova J. (2022). DNA aptamers masking angiotensin converting enzyme 2 as an innovative way to treat SARS-CoV-2 pandemic. *Pharmacol. Res.*.

[61] Song Y., Song J., Wei X. (2020). Discovery of aptamers targeting the receptor-binding domain of the SARS-CoV-2 spike glycoprotein. Anal. Chem..

[62] Schmitz A., Weber A., Bayin M. (2021). A SARS-CoV-2 spike binding DNA aptamer that inhibits pseudovirus infection by an RBD-independent mechanism**. Angew. Chem. Int. Ed..

[63] Chiodi E., Marn A. M., Geib M. T., Ünlü M. S. (2021). The role of surface chemistry in the efficacy of protein and dna microarrays for label-free detection: an overview. *Polymers*.

[64] Vanjur L., Carzaniga T., Casiraghi L. (2021). Copolymer coatings for dna biosensors: effect of charges and immobilization chemistries on yield, strength and kinetics of hybridization. *Polymers*.

[65] Vanjur L., Carzaniga T., Casiraghi L., Chiari M., Zanchetta G., Buscaglia M. (2020). Non-Langmuir kinetics of DNA surface hybridization. Biophys. J..

[66] Mujica M. L., Zhang Y., Bédioui F., Gutiérrez F., Rivas G. (2020). Label-free graphene oxide–based SPR genosensor for the quantification of microRNA21. Anal. Bioanal. Chem..

[67] Xue T., Liang W., Li Y. (2019). Ultrasensitive detection of miRNA with an antimonene-based surface plasmon resonance sensor. *Nat. Commun.*.

[68] Fabri-Faja N., Calvo-Lozano O., Dey P. (2019). Early sepsis diagnosis via protein and miRNA biomarkers using a novel point-of-care photonic biosensor. Anal. Chim. Acta.

[69] Zanchetta G., Carzaniga T., Vanjur L. (2021). Design of a rapid, multiplex, one-pot miRNA assay optimized by label-free analysis. *Biosens. Bioelectron.*.

[70] Lee T., Kwon S., Choi H. J., Lim H., Lee J. (2021). Highly sensitive and reliable microRNA detection with a recyclable microfluidic device and an easily assembled SERS substrate. ACS Omega.

[71] Guselnikova O., Postnikov P., Pershina A., Svorcik V., Lyutakov O. (2019). Express and portable label-free DNA detection and recognition with SERS platform based on functional Au grating. Appl. Surf. Sci..

[72] Qiu G., Gai Z., Tao Y., Schmitt J., Kullak-Ublick G. A., Wang J. (2020). Dual-Functional plasmonic photothermal biosensors for highly accurate severe acute respiratory syndrome coronavirus 2 detection. *ACS Nano*.

[73] Berneschi S., D’Andrea C., Baldini F. (2021). Ion-exchanged glass microrods as hybrid SERS/fluorescence substrates for molecular beacon-based DNA detection. Anal. Bioanal. Chem..

[74] Peter B., Lagzi I., Teraji S. (2018). Interaction of positively charged gold nanoparticles with cancer cells monitored by an in situ label-free optical biosensor and transmission electron microscopy. ACS Appl. Mater. Interfaces.

[75] Masterson A. N., Liyanage T., Kaimakliotis H., Derami H. G., Deiss F., Sardar R. (2020). Bottom-up fabrication of plasmonic nanoantenna-based high-throughput multiplexing biosensors for ultrasensitive detection of microRNAs directly from cancer patients’ plasma. Anal. Chem..

[76] Portela A., Calvo-Lozano O., Estevez M., Escuela A. M., Lechuga L. M. (2020). Optical nanogap antennas as plasmonic biosensors for the detection of miRNA biomarkers. J. Mater. Chem. B.

[77] Špringer T., Song X. C., Ermini M. L., Lamačová J., Homola J. (2017). Functional gold nanoparticles for optical affinity biosensing. Anal. Bioanal. Chem..

[78] Špringer T., Krejčík Z., Homola J. (2021). Detecting attomolar concentrations of microRNA related to myelodysplastic syndromes in blood plasma using a novel sandwich assay with nanoparticle release. *Biosens. Bioelectron.*.

[79] Barroso J., Ortega-Gomez A., Calatayud-Sanchez A. (2020). Selective ultrasensitive optical fiber nanosensors based on plasmon resonance energy transfer. ACS Sens..

[80] Liyanage T., Lai M., Slaughter G. (2021). Label-free tapered optical fiber plasmonic biosensor. *Anal. Chim. Acta*.

[81] Liyanage T., Alharbi B., Quan L., Esquela-Kerscher A., Slaughter G. (2022). Plasmonic-based biosensor for the early diagnosis of prostate cancer. ACS Omega.

[82] Strobbia P., Yang R., Crawford B. M. (2019). Inverse molecular sentinel-integrated fiberoptic sensor for direct and in situ detection of miRNA targets. Anal. Chem..

[83] Jia S., Bian C., Sun J., Tong J., Xia S. (2018). A wavelength-modulated localized surface plasmon resonance (LSPR) optical fiber sensor for sensitive detection of mercury(II) ion by gold nanoparticles-DNA conjugates. Biosens. Bioelectron..

[84] Kim H. M., Uh M., Jeong D. H., Lee Ho Y., Park J. H., Lee S. Ki (2019). Localized surface plasmon resonance biosensor using nanopatterned gold particles on the surface of an optical fiber. Sens. Actuators, B.

[85] Kim H. M., Park J. H., Lee S. Ki (2019). Fiber optic sensor based on ZnO nanowires decorated by Au nanoparticles for improved plasmonic biosensor. *Sci. Rep.*.

[86] Muri H. I., Bano A., Hjelme D. R. (2018). LSPR and interferometric sensor modalities combined using a double-clad optical fiber. *Sensors*.

[87] Wang F., Zhang Y., Lu M. (2021). Near-infrared band Gold nanoparticles-Au film “hot spot” model based label-free ultratrace lead (II) ions detection via fiber SPR DNAzyme biosensor. *Sens. Actuators, B*.

[88] Agrawal N., Saha C., Kumar C. (2020). Detection of L-cysteine using silver nanoparticles and graphene oxide immobilized tapered SMS optical fiber structure. IEEE Sens. J..

[89] Liu K., Zhang J., Jiang J. (2020). Multi-layer optical fiber surface plasmon resonance biosensor based on a sandwich structure of polydopamine-MoSe 2 @Au nanoparticles-polydopamine. Biomed. Opt. Express.

[90] Ngo L. T., Wang W. K., Tseng Y. T. (2021). MutS protein-based fiber optic particle plasmon resonance biosensor for detecting single nucleotide polymorphisms. Anal. Bioanal. Chem..

[91] Che C., Li N., Long K. D. (2019). Activate capture and digital counting (AC + DC) assay for protein biomarker detection integrated with a self-powered microfluidic cartridge. Lab Chip.

[92] Chen Z., Peng Y., Cao Y. (2018). Light-driven nano-oscillators for label-free single-molecule monitoring of MicroRNA. Nano Lett..

[93] Ertsgaard C. T., Wittenberg N. J., Klemme D. J., Barik A., Shih W. C., Oh S. H. (2018). Integrated nanogap platform for sub-volt dielectrophoretic trapping and real-time Raman imaging of biological nanoparticles. Nano Lett..

[94] Sevenler D., Trueb J., Ünlü M. S. (2019). Beating the reaction limits of biosensor sensitivity with dynamic tracking of single binding events. Proc. Natl. Acad. Sci. U. S. A..

[95] Huong V. T., Phuong N. T. T., Tai N. T. (2021). Gold nanoparticles modified a multimode clad-free fiber for ultrasensitive detection of bovine serum albumin. *J. Nanomater.*.

[96] Tseng Y. T., Li W. Y., Yu Y. W. (2020). Fiber optic particle plasmon resonance biosensor for label-free detection of nucleic acids and its application to HLA-B27 mRNA detection in patients with ankylosing spondylitis. *Sensors*.

[97] Lee S. W., Ahmed S., Wang T. (2021). Label-free creatinine optical sensing using molecularly imprinted titanium dioxide-polycarboxylic acid hybrid thin films: a preliminary study for urine sample analysis. *Chemosensors*.

[98] Avci O., Adato R., Ozkumur A. Y., Ünlü M. S. (2016). Physical modeling of interference enhanced imaging and characterization of single nanoparticles. Opt. Express.

[99] Sevenler D., Daaboul G. G., Kanik F. E., Ünlü N. L., Ünlü M. S. (2018). Digital microarrays: single-molecule readout with interferometric detection of plasmonic nanorod labels. ACS Nano.

[100] Priest L., Peters J. S., Kukura P. (2021). Scattering-based light microscopy: from metal nanoparticles to single proteins. Chem. Rev..

[101] Shen H., Tauzin L. J., Baiyasi R. (2017). Single particle tracking: from theory to biophysical applications. Chem. Rev..

[102] Dong J., Maestre D., Conrad-Billroth C., Juffmann T. (2021). Fundamental bounds on the precision of iSCAT, COBRI and dark-field microscopy for 3D localization and mass photometry. *J. Phys. D: Appl. Phys.*.

[103] Enoki S., Iino R., Morone N. (2012). Label-free single-particle imaging of the influenza virus by objective-type total internal reflection dark-field microscopy. *PLoS One*.

[104] Chen S., Huang Z., Liu C. (2022). Label free imaging and deep tracking of single biological nanoparticles in free solution by reflection enhanced dark field scattering microscopy. *Sens. Actuators B Chem.*.

[105] Gopalan D., Nair P. R. (2020). Dynamic tracking biosensors: finding needles in a haystack. ACS Sens..

[106] Goldfain A. M., Garmann R. F., Jin Y., Lahini Y., Manoharan V. N. (2016). Dynamic measurements of the position, orientation, and DNA content of individual unlabeled bacteriophages. J. Phys. Chem. B.

[107] Huang Y. F., Zhuo G. Y., Chou C. Y., Lin C. H., Chang W., Hsieh C. L. (2017). Coherent brightfield microscopy provides the spatiotemporal resolution to study early stage viral infection in live cells. ACS Nano.

[108] Taylor R. W., Sandoghdar V. (2019). Interferometric scattering microscopy: seeing single nanoparticles and molecules via Rayleigh scattering. Nano Lett..

[109] Ozkumur E., Needham J. W., Bergstein D. A. (2008). Label-free and dynamic detection of biomolecular interactions for high-throughput microarray applications. *Proc. Natl. Acad. Sci. U.S.A.*.

[110] Špačková B., Moberg H. K., Fritzsche J. (2022). Label-free nanofluidic scattering microscopy of size and mass of single diffusing molecules and nanoparticles. *Nat. Methods*.

[111] Kashkanova A. D., Blessing M., Gemeinhardt A., Soulat D., Sandoghdar V. (2022). Precision size and refractive index analysis of weakly scattering nanoparticles in polydispersions. Nat. Methods.

[112] Cole D., Young G., Weigel A., Sebesta A., Kukura P. (2017). Label-free single-molecule imaging with numerical-aperture-shaped interferometric scattering microscopy. ACS Photonics.

[113] Zhang P., Ma G., Wan Z., Wang S. (2021). Quantification of single-molecule protein binding kinetics in complex media with prism-coupled plasmonic scattering imaging. ACS Sens..

[114] Yu X. C., Tang S. J., Liu W. (2022). Single-molecule optofluidic microsensor with interface whispering gallery modes. *Proc. Natl. Acad. Sci.*.

[115] Subramanian S., Jones H. B. L., Frustaci S. (2021). Sensing enzyme activation heat capacity at the single-molecule level using gold-nanorod-based optical whispering gallery modes. *ACS Appl. Nano Mater.*.

[116] Mauranyapin N. P., Madsen L. S., Taylor M. A., Waleed M., Bowen W. P. (2017). Evanescent single-molecule biosensing with quantum-limited precision. Nat. Photonics.

[117] Zhang P., Ma G., Dong W., Wan Z., Wang S., Tao N. (2020). Plasmonic scattering imaging of single proteins and binding kinetics. Nat. Methods.

[118] Maccaferri N., Barbillon G., Koya A. N., Lu G., Acuna G. P., Garoli D. (2021). Recent advances in plasmonic nanocavities for single-molecule spectroscopy. Nanoscale Adv..

[119] Frustaci S., Vollmer F. (2019). Whispering-gallery mode (WGM) sensors: review of established and WGM-based techniques to study protein conformational dynamics. Curr. Opin. Chem. Biol..

[120] Subramanian S., Vincent S., Frank V. (2020). Effective linewidth shifts in single-molecule detection using optical whispering gallery modes. Appl. Phys. Lett..

[121] Li H., Huang Y., Hou G. (2019). Single-molecule detection of biomarker and localized cellular photothermal therapy using an optical microfiber with nanointerface. *Sci. Adv.*.

[122] Vörös J. (2004). The density and refractive index of adsorbing protein layers. Biophys. J..

